# Critical Assessment of Short-Read Assemblers for the Metagenomic Identification of Foodborne and Waterborne Pathogens Using Simulated Bacterial Communities

**DOI:** 10.3390/microorganisms10122416

**Published:** 2022-12-06

**Authors:** Zhao Chen, Jianghong Meng

**Affiliations:** 1 Joint Institute for Food Safety and Applied Nutrition, University of Maryland, College Park, MD 20742, USA; 2 Center for Food Safety and Security Systems, University of Maryland, College Park, MD 20742, USA; 3 Department of Nutrition and Food Science, University of Maryland, College Park, MD 20742, USA

**Keywords:** Illumina sequencing, metagenome assembly, short-read assembler, foodborne pathogen, waterborne pathogen, spinach, surface water

## Abstract

Metagenomics offers the highest level of strain discrimination of bacterial pathogens from complex food and water microbiota. With the rapid evolvement of assembly algorithms, defining an optimal assembler based on the performance in the metagenomic identification of foodborne and waterborne pathogens is warranted. We aimed to benchmark short-read assemblers for the metagenomic identification of foodborne and waterborne pathogens using simulated bacterial communities. Bacterial communities on fresh spinach and in surface water were simulated by generating paired-end short reads of Illumina HiSeq, MiSeq, and NovaSeq at different sequencing depths. Multidrug-resistant *Salmonella* Indiana SI43 and *Pseudomonas aeruginosa* PAO1 were included in the simulated communities on fresh spinach and in surface water, respectively. ABySS, IDBA-UD, MaSuRCA, MEGAHIT, metaSPAdes, and Ray Meta were benchmarked in terms of assembly quality, identifications of plasmids, virulence genes, *Salmonella* pathogenicity island, antimicrobial resistance genes, chromosomal point mutations, serotyping, multilocus sequence typing, and whole-genome phylogeny. Overall, MEGHIT, metaSPAdes, and Ray Meta were more effective for metagenomic identification. We did not obtain an optimal assembler when using the extracted reads classified as *Salmonella* or *P. aeruginosa* for downstream genomic analyses, but the extracted reads showed consistent phylogenetic topology with the reference genome when they were aligned with *Salmonella* or *P. aeruginosa* strains. In most cases, HiSeq, MiSeq, and NovaSeq were comparable at the same sequencing depth, while higher sequencing depths generally led to more accurate results. As assembly algorithms advance and mature, the evaluation of assemblers should be a continuous process.

## 1. Introduction

In recent years, metagenomics has become a powerful tool for recovering individual genomes of foodborne and waterborne pathogens directly from complex microbiota to identify both culturable and unculturable microorganisms with meaningful implications [1]. Currently, Illumina platforms with high accuracy (~0.1% of base-calling errors) and short read lengths (75–300 bp) are widely used in most shotgun metagenomic sequencing studies due to the low DNA input requirements and high sequencing throughput [2]. Metagenome assembly typically succeeds in merging many of the reads and results in more contiguous sequences (contigs), depending on the sequencing depth and the complexity of the microbial species [3]. However, the short length of Illumina reads often results in highly fragmented sequences of de novo assemblies, which reflects their inability to assemble repetitive regions longer than the read length [4]. This fragmentation can be magnified when handling microbiota with repeats shared by multiple taxa [5]. While it is possible to use partial metagenomic sequences to perform pathogen identification to fully uncover the genetic potential, the extended genomic regions or completely restored genomes from the microbiome should be further analyzed. The identification of positions and structures of certain genes, such as antimicrobial resistance genes (ARGs) within a metagenome, becomes more viable only if Illumina short reads are assembled into longer sequence stretches [6]. Meanwhile, metagenome assembly also removes some sequencing errors through consensus, though this can introduce new assembly errors that may potentially affect downstream analyses [6].

Compared to genome assembly, metagenome assembly for a food and water sample is more complicated and may require special-purpose assembly algorithms [7]. The most challenging issue could be that bacterial communities in complex matrices normally contain related species or sub-species in different—and unknown—abundances, producing extensive inter-genomic overlaps that hinder the assembly process [8]. The sequencing depth of a particular species is rarely high unless it is present in high quantities [9]. A second obstacle that makes metagenome assembly more difficult is the highly uneven sequencing depth of different microorganisms within the sample, which obstructs the algorithm in resolving repeats and removing erroneous reads [10].

While metagenome assembly is still a field in its infancy relative to genome assembly, there has recently been an increasing interest in the development of new assemblers [9]. With the highly dynamic evolvement of assembly algorithms applicable to Illumina short reads, a great effort has been made to benchmark assemblers suitable for short-read-based shotgun metagenomics. Unfortunately, available assemblers are often compared only concerning assembly quality statistics [11]. From the perspective of food safety and public health, the overall performance of metagenome assembly on downstream analyses is, however, often neglected, especially regarding the identification of foodborne and waterborne pathogens. No well-defined microbial community standards mimicking food or water microbiomes containing bacterial pathogens with significant genotypic characteristics, such as multidrug resistance and plasmids, are commercially available to allow researchers to validate and fine-tune bioinformatic workflows for food and water microbiome research. This greatly impedes the comparability of metagenome assemblers for food and water microbiomes. Therefore, while arriving at the mock communities of food and water microbiomes remains challenging, simulated communities generated *in silico*, in which pathogens of interest are accurately characterized, should still be used as a defined input to assess the performance of metagenome assemblers in benchmarking studies on food and water microbiomes containing bacterial pathogens. The results of this initiative will provide a valuable universal benchmarking for the metagenomic identification of bacterial pathogens in food and water microbiomes, as well as an important evaluation of different assemblers’ capabilities to reliably reconstruct their genomes.

In this research, we benchmarked six short-read assemblers designed for genomics or metagenomics using simulated bacterial communities on fresh spinach and in surface water based on quality features and attributes, which would inevitably influence the selection of a suitable assembler. Most importantly, we primarily studied whether the assembly process could improve downstream metagenomic identification of foodborne and waterborne pathogens in a metagenomic context. *Salmonella* Indiana caused salmonellosis in 27 people who consumed a contaminated Waldorf salad containing leafy greens served in a cold buffet in the Netherlands in 1981 [12]. Dole Fresh Vegetables, Inc. and Vegpro International recalled baby spinach due to potential contamination with *Salmonella* in 2019 and 2020, respectively [13,14]. According to van Asperen et al. [15], *Pseudomonas aeruginosa* is the major cause of otitis externa in surface water swimmers. Therefore, in the present study, *S.* Indiana and *P. aeruginosa* were included in the simulated communities on fresh spinach and in surface water, respectively. Additionally, we also compared Illumina HiSeq, MiSeq, and NovaSeq with different numbers of reads to see how sequencing platforms and depths could influence downstream analyses.

## 2. Materials and Methods

### 2.1. Reference Datasets

The simulated bacterial community on fresh spinach was built based on the published data by Lopez-Velasco et al. [16], which contained five phyla, including actinobacteria, acidobacteria, deinococcus-thermus, firmicutes, and proteobacteria (alpha-proteobacteria, gamma-proteobacteria, and beta-proteobacteria) (Table S1). The simulated bacterial community in surface water (urban river water) contained six phyla, including actinobacteria, acidobacteria, proteobacteria (alpha-proteobacteria, gamma-proteobacteria, and beta-proteobacteria), bacteroidetes, planctomycetes, and verrucomicrobia [17] (Table S2). The reference genome of the representative microorganism for each family was obtained from the National Center for Biotechnology Information (NCBI) Reference Sequence (RefSeq) Database. Multidrug-resistant *S.* Indiana SI43 reported in our previous study [18] was included in the community on fresh spinach to represent the family of Enterobacteriaceae, while multidrug-resistant *P. aeruginosa* PAO1 was used to represent the order of Pseudomonadales in the community in surface water. The relative abundance of each family was normalized to have a total relative abundance of 100% to allow subsequent analyses to focus solely on classified reads by excluding unclassified and eukaryotic reads.

### 2.2. Illumina Short-Read Simulation

To assess the effects of sequencing platforms and depths on metagenome assembly, Illumina paired-end short reads were simulated using InSilicoSeq 1.5.4 [19] with pre-computed error models of three Illumina sequencing platforms, including HiSeq (2 × 125 bp), MiSeq (2 × 300 bp), and NovaSeq (2 × 150 bp). One million reads of each error model were generated for the community on fresh spinach, with the relative abundance of the input genomes drawn from a log-normal distribution. Additionally, 2.4 million HiSeq and 2 million NovaSeq reads were produced to compare the platforms with MiSeq at the same sequencing depth. The number of MiSeq reads was then increased to 1.5 million to examine if a higher sequencing depth could improve the downstream metagenomic analyses. Regarding the community in surface water, 2.4 million HiSeq, 1.5 million MiSeq, and 2 million NovaSeq reads were first simulated. Afterward, to compare three platforms at the same sequencing depth, the numbers of HiSeq, MiSeq, and NovaSeq reads were then increased to 4.8, 2, and 4 million, respectively.

### 2.3. Metagenome Assembly

In this study, we benchmarked six widely used short-read assemblers, including ABySS 2.2.3 [20], IDBA-UD 1.1.3 [21], MaSuRCA 4.0.9 [22], MEGAHIT 1.2.9 [23], metaSPAdes 3.15.4 [24], and Ray Meta 2.3.1 [25]. ABySS and Ray Meta were subjected to default *k*-mer sizes of 41 and 21 bp, respectively. To assemble the simulated reads with MaSuRCA, *k*-mer size was automatically computed between 25 and 127 bp based on the read data and GC content (%). IDBA-UD was run by iterating *k*-mer size from 20 to 100 bp with an increment of 20 bp. For assemblers with multi-*k*-mer capabilities, including MEGAHIT and MetaSPAdes, assembly processes were run with default settings, with *k*-mer sizes of 21, 29, 39, 59, 79, 99, 119, and 141 bp, and *k*-mer sizes of 21, 33, and 55 bp, respectively.

After metagenome assembly, the taxon of each sequence was identified using Kraken 2 2.1.2 by querying the standard database [26], with a *k*-mer size of 35 bp, a minimizer length of 35 bp, and a minimizer space of 6 bp, and two overlapping *k*-mers sharing the same minimizer needed to make a call (minimum hit groups). Sequences specifically assigned to *Salmonella* (taxonomy ID: 590) or *Pseudomonas* (taxonomy ID: 286) were pooled from each assembly using the read extraction module of Kraken 2.

### 2.4. Assessment of Assembly Quality

The quality of each metagenome assembly was assessed using QUAST 5.0.2 [27] with the MetaQUAST extension [28] by generating relevant metrics, including the number of contigs, the length of the largest contig (bp), total length (bp), N50, and L50. For the extracted reads classified as *Salmonella* or *Pseudomonas* in each metagenome assembly, the performance of assemblers was assessed by aligning sequences to the reference genome of *S.* Indiana SI43 or *P. aeruginosa* PAO1, respectively. The GC content (%) and genome fraction (%) of extracted reads, as well as the numbers of misassemblies, N’s per 100 kbp, mismatches per 100 kbp, and indels per 100 kbp, were also computed. The completeness of each assembly was quantitatively assessed by performing a benchmarking universal single-copy ortholog (BUSCO) analysis using BUSCO 5.2.2 [29] according to the expected gene content of an assembly and length alignments to the BUSCO profiles, with 0.001 as the E-value cutoff for BLAST searches, three candidate regions to consider, and Prodigal 2.6.3 as the gene predictor. The best lineage dataset (bacteria_odb10: the number of genomes, 4085; the number of BUSCOs, 124) was automatically selected with the automated lineage selection process based on phylogenetic placement. The degree of genome completeness was expressed as BUSCO scores, including complete, fragmented, and missing BUSCOs that indicate the fractions of high-identity full-length genes, partially present genes, and absent genes, respectively.

### 2.5. Identifications of Plasmids, Virulence Genes, Salmonella Pathogenicity Island (SPI), ARGs, and Chromosomal Point Mutations

Plasmids were detected using staramr 0.7.2 [30] against known plasmid sequences in the PlasmidFinder database [31], with 98% minimum identity and 60% minimum coverage. Virulence genes were detected by comparing with known gene sequences using ABRicate 1.0.1 (https://github.com/tseemann/abricate; accessed on 5 December 2022) integrated with the Virulence Factors Database (VFDB) [32], with an 80% minimum identity and 60% minimum coverage. SPIFinder 2.0 [33] was applied to identify SPI, with 95% minimum identity and 60% minimum coverage. ARGs and chromosomal point mutations were identified using staramr 0.7.2 against known ARG and point mutation sequences in the ResFinder [34] and PointFinder [35] databases, respectively, with 98% minimum identity and 60% minimum coverage. Contigs carrying ARGs were then searched using the NCBI nucleotide BLAST with the MegaBLAST algorithm [36] for highly similar sequences and the nr/nt database to see if *S.* Indiana SI43 or *P. aeruginosa* PAO1 was among the blast results, with 100 as the maximum target sequences, 0.05 as the expect threshold, 28 as the word size, 1, −2 as the match/mismatch scores, respectively, linear gap costs, low-complexity filter, and lookup-table-only mask.

### 2.6. Serotyping

*Salmonella* serotype was predicted from the extracted reads using SISTR 1.1.1, whose prediction algorithm is based on O (somatic) and H (flagellar) antigens and/or serogroup-specific probes [37]. PAst 1.0 was used for the serotyping of *P. aeruginosa* based on the analysis of the O-specific antigen [38].

### 2.7. Multilocus Sequence Typing (MLST)

MLST was conducted using mlst 2.19.0 with incorporated components of the PubMLST database [39] by scanning the extracted reads against traditional PubMLST typing schemes based on seven housekeeping genes. The MLST scheme had a minimum identity of the full allele of 95%, a minimum coverage of the partial allele of 10%, and a minimum score to match a scheme of 50.

### 2.8. Whole-Genome Phylogenetic Analyses

To examine how they performed in the whole-genome phylogenetic inference, the extracted reads classified as *Salmonella* in the community on fresh spinach were compared with *S.* Indiana SI43 and 20 *S.* Indiana strains (Table S3) or 20 *Salmonella* strains of different species and serotypes (Table S4), while the extracted reads classified as *Pseudomonas* in the community in surface water were compared with *P. aeruginosa* PAO1 and 30 *P. aeruginosa* strains (Table S5). CSI Phylogeny 1.4 was used to call SNPs and infer phylogeny based on the concatenated alignment of the high-quality SNP [40], with 10 bp as the minimum distance between SNPs, 30 as the minimum SNP quality, 10× as the minimum depth at SNP positions, 10% as the minimum relative depth at SNP positions, 25 as the minimum read mapping quality, and 1.96 as the minimum Z-score. *S.* Typhimurium LT2 (RefSeq assembly accession: GCF_000006945.2) and *P. aeruginosa* DSM 50071 (RefSeq assembly accession: GCF_001045685.1) served as the reference genomes for the whole-genome phylogenetic analyses of *Salmonella* and *P. aeruginosa*, respectively.

## 3. Results

### 3.1. Assembly Quality

According to the data obtained when 1 million HiSeq reads representing the bacterial community on fresh spinach were assembled, ABySS, MaSuRCA (Table 1), and Ray Meta were the least robust, whereas metaSPAdes had the best assembly strategy, closely followed by IDBA-UD and MEGAHIT. metaSPAdes produced an assembly that had the greatest number of contigs, length of the largest contig, total length, and N50 relative to other assemblers. Overall, IDBA-UD, MEGAHIT, metaSPAdes, and Ray Meta performed similarly in assembling 1 million MiSeq reads in terms of all quality parameters evaluated. Concerning 1 million NovaSeq reads, metaSPAdes performed the best in terms of the length of the largest contigs and total length, while IDBA-UD produced the highest N50 and Ray Meta produced the greatest number of contigs and highest L50. ABySS or MaSuRCA did not perform well when assembling MiSeq and NovaSeq reads, which agrees with their performance when HiSeq reads were assembled. Altogether, metaSPAdes was observed to be able to effectively assemble reads of different sequencing platforms. The quality of the IDBA-UD and MEGAHIT assemblies was close, while the performance of Ray Meta was moderate. ABySS or MaSuRCA did not generate an acceptable assembly. When 1 million reads were assembled, compared to the ABySS, MaSuRCA, and Ray Meta assemblies, the complete BUSCOs of the IDBA-UD, MEGAHIT, and metaSPAdes assemblies were higher, whereas their fragmented and missing BUSCOs were lower.

As expected, when the numbers of HiSeq and NovaSeq reads representing the bacterial community on fresh spinach were increased to 2.4 and 2 million, respectively, the overall performance of each assembler was improved. In contrast to when 1 million reads were assembled, all assemblers performed comparably in terms of the quality parameters determined. ABySS, metaSPAdes, and Ray Meta generated more contigs compared to other assemblers. MEGAHIT, metaSPAdes, and Ray Meta produced assemblies with greater total lengths, while the IDBA-UD, MaSuRCA, and Ray Meta assemblies had greater lengths of the largest contig and N50s. Regarding the sequencing platforms at the same sequencing depth, overall, assemblies of MiSeq reads had higher numbers of contigs, greater total lengths, and greater L50s, whereas assemblies of HiSeq reads had greater lengths of the largest contig and N50s. NovaSeq reads performed moderately compared to HiSeq and MiSeq reads. Large gains in genome completeness were observed when the sequencing depths of HiSeq and NovaSeq were increased, where the complete BUSCOs of the ABySS, MaSuRCA, and Ray Meta assemblies increased, while the fragmented and missing BUSCOs decreased correspondingly. The number of MiSeq reads was then increased to 1.5 million to see whether a higher sequencing depth could further improve assembly quality. With the increase in the sequencing depth, the numbers of contigs, lengths of the largest contig, total length, and N50s of all assemblies increased except for the ABySS assembly, whose number of contigs decreased from 38,624 to 27,450; however, their L50s decreased when the sequencing depth was increased. Compared to when 1 million MiSeq reads were assembled, the ABySS, MaSuRCA, and Ray Meta assemblies of 1.5 million MiSeq reads yielded higher complete BUSCOs, and lower fragmented and missing BUSCOs.

Noticeably, when the simulated reads of the bacterial community in surface water were tested, the quality of the assemblies generated by different assemblers was similar, regardless of sequencing platform and depth (Table 2). The complete BUSCOs of all assemblies of 2.4 million HiSeq reads, 1.5 million MiSeq reads, and 2 million NovaSeq reads were 100%, irrespective of assembler, with only one exception where the Ray Meta assemblies had lower complete BUSCOs, and higher fragmented and missing BUSCOs. When the sequencing depths of HiSeq and MiSeq were increased to 4.8 million and 2 million, respectively, the complete BUSCOs of the Ray Meta assemblies increased to 100%, while their fragmented and missing BUSCOs decreased to 0.0%. In contrast, the complete and fragmented BUSCOs of the Ray Meta assembly of 4 million NovaSeq reads were 99.2% and 0.8%, respectively. At the same sequencing depth, the completeness of all assemblies was comparable, regardless of assembler.

We then assessed the quality of the extracted reads classified as *Salmonella* and *Pseudomonas* in the fresh spinach and the surface water metagenome assemblies with different sequencing depths, respectively (Tables S3 and S4). Overall, the Ray Meta assemblies had a relatively higher metagenome quality compared to others, which had greater numbers of contigs and total lengths but fewer N’s, mismatches, and indels per 100 kbp, irrespective of bacterial community and sequencing platform and depth. For the extracted reads classified as Salmonella in the fresh spinach metagenome assemblies, overall, MiSeq reads produced assemblies with higher numbers of contigs, and greater total lengths, N50s, L50s, and genome fractions compared to HiSeq and NovaSeq at the same sequencing depth. The assemblies of HiSeq reads were more accurate than MiSeq and NovaSeq, with lower numbers of misassemblies, N’s per 100 kbp, mismatches per 100 kbp, and indels per 100 kbp. For the extracted reads classified as Pseudomonas in the surface water metagenome assemblies, the overall assembly quality of HiSeq, MiSeq, and NovaSeq reads was similar.

### 3.2. Plasmids

Three plasmids, including IncHI2A, IncHI2, and IncQ1, were present in *S.* Indiana SI43, but none was detected in *P. aeruginosa* PAO1. Plasmids were not identified in any of the other representative microorganisms of the simulated bacterial community of fresh spinach or surface water. For the spinach metagenome assemblies of 1 million reads (Table 3), IDBA-UD, MEGAHIT, and metaSPAdes assemblies showed plasmid profiles that were congruent with the reference genome, regardless of the sequencing platform. The ABySS assembly of MiSeq reads and the MaSuRCA assembly of NovaSeq reads also contained plasmids that were consistent with the reference genome, whereas none of the Ray Meta assemblies displayed an accurate plasmid pattern. When the number of HiSeq and NovaSeq reads was increased to 2.4 and 2 million, respectively, the plasmids present in the reference genome were detected in all assemblies, while the ABySS and Ray Meta assemblies failed to recover these plasmids. It is worth noting that IncHI2 identified in the MaSuRCA assembly of 1 million NovaSeq reads was absent in that with 2 million NovaSeq reads. HiSeq, MiSeq, and NovaSeq performed similarly at the same sequencing depth. None of the plasmids were present in the extracted reads classified as *Salmonella* in the fresh spinach metagenome assemblies.

### 3.3. ARGs

None of the spinach metagenome assemblies of 1 million HiSeq reads provided AMR profiles that agree with the reference genome (Tables S5–S7). MEGAHIT and metaSPAdes were superior to other assemblers and produced assemblies with AMR patterns similar to the reference genome. ABySS performed the worst and did not produce an assembly with ARGs. None of the assemblies carried point mutation in *gyrA* or *parC* associated with nalidixic acid resistance. MaSuRCA, MEGAHIT, and metaSPAdes were the most effective assemblers for assembling 1 million MiSeq reads, whose assemblies had AMR profiles similar to the reference genome. Ray Meta performed the worst and did not generate an assembly with ARGs conferring resistance to gentamicin, ciprofloxacin I/R, trimethoprim, chloramphenicol, amikacin, tetracycline, and nalidixic acid. For 1 million NovaSeq reads, IDBA-UD, MEGAHIT, and metaSPAdes were comparable for the AMR analysis and performed the best among all assemblers, whose assemblies showed predicted phenotypes consistent with the reference genome. The MaSuRCA and Ray Meta assemblies failed to provide accurately predicted phenotypes, whereas the ABySS assembly only carried *ARR-3* and *sul1* associated with rifampicin and sulfisoxazole resistance, respectively. When the number of MiSeq reads was increased to 1.5 million (Tables S8–S10), all assemblers produced assemblies with predicted phenotypes congruent with the reference, indicating that they were all capable of acquiring assemblies that can be used for accurate predictions of AMR phenotypes, although a point mutation in *gyrA* associated with nalidixic acid resistance was still absent in the IDBA-UD and Ray Meta assemblies. When the numbers of HiSeq and NoveSeq reads were increased to 2 and 2.4 million (Tables S11–S13), respectively, the ABySS, IDBA-UD, MEGAHIT, and metaSPAdes assemblies provided predicted phenotypes consistent with the reference genome. Regarding the assembly of 2.4 million HiSeq reads, the ARG associated with trimethoprim resistance, *dfrA12*, was absent in the MaSuRCA assembly, while the point mutation in *gyrA* or *parC* responsible for nalidixic acid resistance was not identified in the Ray Meta assembly. When 2 million NovaSeq reads were assembled, *blaTEM-34* and *blaTEM-30* that were absent in the reference were identified in the MaSuRCA and Ray Meta assemblies, resulting in inaccurate phenotype prediction of amoxicillin/clavulanic acid resistance. Nonetheless, with higher HiSeq, MiSeq, and NovaSeq sequencing depths, the assemblies still did not carry the ARGs present in *Micrococcus luteus* AS2, *Rhizobium* sp. S41, or *Stenotrophomonas maltophilia* NCTC10258. Interestingly, *cmx* in *M. luteus* AS2 that was detected in the MEGAHIT and metaSPAdes assemblies of 1 million MiSeq reads was not present in any of the assemblies of 2.4 million HiSeq, 1.5 million MiSeq, and 2 million NovaSeq reads. Overall, HiSeq and NovaSeq outperformed MiSeq at the same sequencing depth. For instance, significantly more ARGs were identified in the Ray Meta assembly of 2.4 million HiSeq, and 2 million NovaSeq reads compared to 1 million MiSeq reads.

For the surface water metagenome, the ABySS or Ray Meta assemblies of 1.5 million MiSeq reads did not carry any ARGs, while metaSPAdes performed the best, followed by IDBA-UD and MaSuRCA (Tables S14 and S15). The IDBA-UD, MaSuRCA, and metaSPAdes assemblies led to consistently predicted phenotypes, none of which harbored *aph(3′)-IIb* related to kanamycin resistance. Only one ARG in *P. norimbergensis* DSM 11628, *blaOXA-157*, was identified in the MEGAHIT assembly. *aph(3′)-IIb* or *blaOXA-50* was not detected in any of the assemblies of 1.5 million MiSeq reads. None of the assemblies of 2.4 million HiSeq and 2 million NovaSeq reads performed well and only contained a few ARGs (Tables S16 and S17). When the sequencing depth of HiSeq was increased to 4.8 million, *fosA* was the only ARG in *P. aeruginosa* PAO1 that could be detected in the ABySS, IDBA-UD, and MEGAHIT assemblies, while the IDBA-UD, MEGAHIT, and metSPAdes assemblies harbored *blaOXA-157* in *P. norimbergensis* DSM 11628 and *erm(E)* in *S. erythraea* NRRL 2338. *aph(3′)-IIb*, *blaOXA-50*, *blaPAO*, or *catB7* was not detected in any of the assemblies (Tables S18 and S19). None of the ARGs were present in the MaSuRCA or Ray Meta assembly of 4.8 million HiSeq reads. ABySS, MaSuRCA, or Ray Meta did not produce an assembly of 2 million MiSeq reads that contained any ARGs. Although the genotypes of the IDBA-UD, MEGAHIT, and metaSPAdes assemblies were comparable, the predicted phenotype of the metaSPAdes assembly was consistent with that of the reference genome. *blaPAO* was absent in all assemblies of 4 million NovaSeq reads. Ray Meta did not perform well, with only *erm(E)* detected but not confirmed to be originated from *S. erythraea* NRRL 2338. metaSPAdes produced the only assembly that carried *fosA* and showed an accurately predicted phenotype, while IDBA-UD and MEGAHIT were comparable, closely followed by ABySS and MaSuRCA. Noticeably, NovaSeq performed significantly better than HiSeq and MiSeq at the same sequencing depth for AMR profiling.

The extracted reads classified as *Salmonella* in the fresh spinach metagenome assemblies and *Pseudomonas* from the surface water metagenome assemblies performed poorly and failed to exhibit any accurate AMR profile (Tables S20–S30).

### 3.4. Virulence Genes and SPIs

The representative microorganisms of the simulated bacterial community of fresh spinach or surface water did not carry any virulence genes except *S.* Indiana SI43 and *P. aeruginosa* PAO1, which contained 91 and 241 virulence genes, respectively. Relative to *S.* Indiana SI43, the numbers of virulence genes in the spinach metagenome assemblies of 1 million HiSeq and NovaSeq reads were not accurate, although IDBA-UD, MEGAHIT, and metaSPAdes assemblies of NovaSeq reads contained 90 virulence genes (Table 4). In contrast, the numbers of virulence genes in the IDBA-UD, MEGAHIT, and metaSPAdes assemblies of 1 million MiSeq reads were consistent with *S.* Indiana SI43. Noticeably, when the sequencing depths of HiSeq and NovaSeq were increased, more virulence genes were identified in their assemblies, but the numbers of virulence genes were still significantly different from that in *S.* Indiana SI43. It should also be noted that the numbers of virulence genes in the IDBA-UD, MEGAHIT, and metaSPAdes assemblies of NovaSeq reads increased from 90 to 124, 124, and 126, respectively, when the numbers of reads were increased. As a comparison, the assemblies of 1.5 million MiSeq reads produced similar numbers of virulence genes to *S.* Indiana SI43, irrespective of assembler, though the numbers of virulence genes in the IDBA-UD and metaSPAdes assemblies became inaccurate (93 and 89, respectively).

Concerning the identification of virulence genes in the surface water metagenome assemblies, none of the assemblers could produce assemblies with the numbers of virulence genes similar to *P. aeruginosa* PAO1, regardless of the sequencing platform (Table 5). Nevertheless, it is noteworthy that increasing the numbers of HiSeq, MiSeq, and NovaSeq reads to 4.8, 2, and 4 million, respectively, greatly improved the identification of virulence genes in all assemblies, although they still failed to display the numbers of virulence genes consistent with *P. aeruginosa* PAO1. IDBA-UD, MEGAHIT, and metaSPAdes outperformed other assemblers in all cases, irrespective of sequencing platform and depth.

None of the spinach metagenome assemblies of 1 million reads yielded an accurate SPI profile, regardless of the sequencing platform (Table 6). The IDBA-UD and metaSPAdes assemblies of MiSeq reads had SPI patterns that were most similar to *S.* Indiana SI43, in which only one SPI-3 and one SPI-4 were absent, respectively. When the numbers of HiSeq and NovaSeq reads were increased, the performance of each assembler greatly improved, with the IDBA-UD and metaSPAdes assemblies of HiSeq reads, and the metaSPAdes assembly of NovaSeq reads showing accurate SPI patterns. Overall, MiSeq performed better compared to HiSeq and NovaSeq at the same sequencing depth.

The extracted reads classified as *Salmonella* or *P. aeruginosa* did not perform well and showed significantly different numbers of virulence genes from the reference genome (Tables S31 and S32). None of the extracted reads classified as *Salmonella* in the fresh spinach metagenome assemblies of 1 million reads, 2.4 million HiSeq reads, or 2 million NovaSeq reads displayed an accurate SPI profile or carried SPIs similar to *S.* Indiana SI43 (Table S33). By comparison, when the number of MiSeq reads was increased to 1.5 million, the extracted reads classified as *Salmonella* from the ABySS, MaSuRCA, and metaSPAdes assemblies harbored all SPIs in *S.* Indiana SI43.

### 3.5. Salmonella Serotypes

The extracted reads classified as *Salmonella* in the assemblies of 1 and 2.4 million HiSeq reads, as well as 1 and 2 million NovaSeq reads, were accurately serotyped (Table S34). ABySS, MEGAHIT, and Ray Meta produced assemblies of 1 million MiSeq reads serotyped as Indiana. Interestingly, when the number of MiSeq reads was increased to 1.5 million, Ray Meta was the only assembler that provided accurate serotyping.

### 3.6. MLST

None of the extracted reads classified as *Salmonella* in the fresh spinach metagenome assemblies of 1 million reads provided an accurate MLST result except the Ray Meta assemblies of HiSeq and MiSeq reads (Table S35). When the numbers of HiSeq and NovaSeq reads were increased to 2.4 and 2 million, respectively, the extracted reads from the Ray Meta assembly of HiSeq reads were unambiguously typed, while the extracted reads from the NovaSeq assemblies failed to produce an accurate MLST result. Only the extracted reads from the Ray Meta assembly of 1.5 million MiSeq reads were accurately typed. The extracted reads classified as *Pseudomonas* from the surface water metagenome assemblies did not provide accurate MLST results, regardless of sequencing platform and depth, and assembler.

### 3.7. Whole-Genome Phylogeny

**Figure 1:**
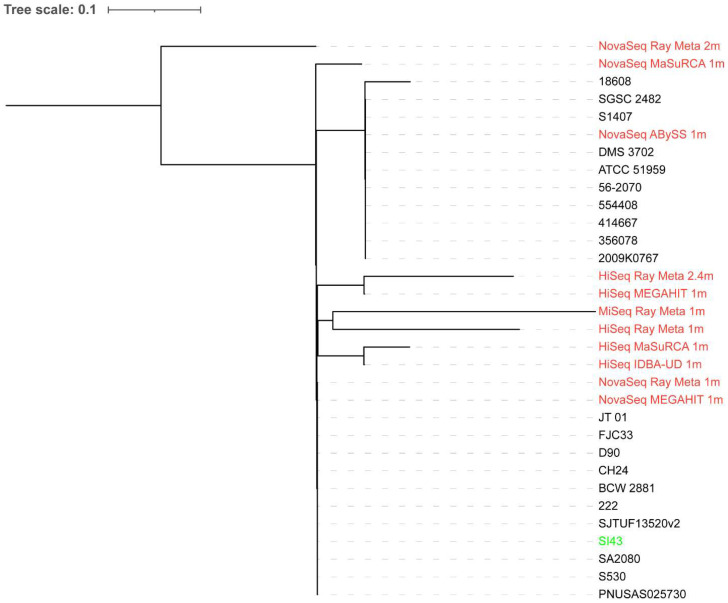
Whole-genome phylogenetic tree of extracted reads classified as *Salmonella* from the spinach metagenome assemblies in addition to *S.* Indiana SI43 compared to 20 *S.* Indiana strains. The scale bar indicates the genetic distance. Assemblies are indicated in red, while *S.* Indiana SI43 is indicated in green.

**Figure 2:**
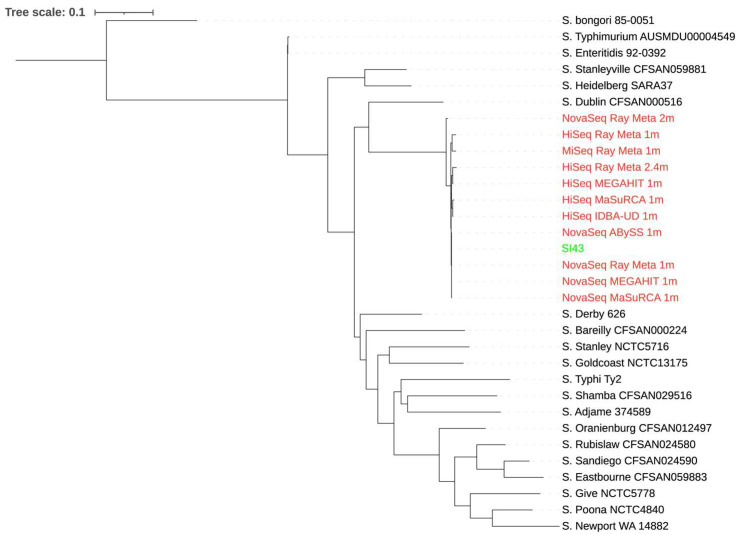
Whole-genome phylogenetic tree of extracted reads classified as *Salmonella* from the spinach metagenome assemblies in addition to *S.* Indiana SI43 compared to 20 *Salmonella* strains of different species and serotypes. The scale bar indicates the genetic distance. Assemblies are indicated in red, while *S.* Indiana SI43 is indicated in green.

A total of 20 *S.* Indiana strains were used for the phylogenetic analysis of the extracted reads classified as *Salmonella* from the spinach metagenome assemblies. Some extracted reads from the assemblies with small total lengths were not included due to an error produced by CSI Phylogeny when they were aligned with others. As shown in Figure 1, the extracted reads from the assemblies did not produce a phylogenetic tree topology where they clustered together with *S.* Indiana SI43. Although biased results were observed across the extracted reads from all assemblies, those from the MEGAHIT assembly of 1 million NovaSeq reads showed the smallest distance from *S.* Indiana SI43. In contrast, when 20 *Salmonella* strains of different species and serotypes were used for the phylogenetic analysis (Figure 2), the extracted reads from the assemblies formed a single monophyletic clade with *S.* Indiana SI43, irrespective of sequencing platform and depth, and assembler. The extracted reads from the ABySS and Ray Meta assemblies of 1 million NovaSeq reads had the smallest distance from *S.* Indiana SI43.

**Figure 3:**
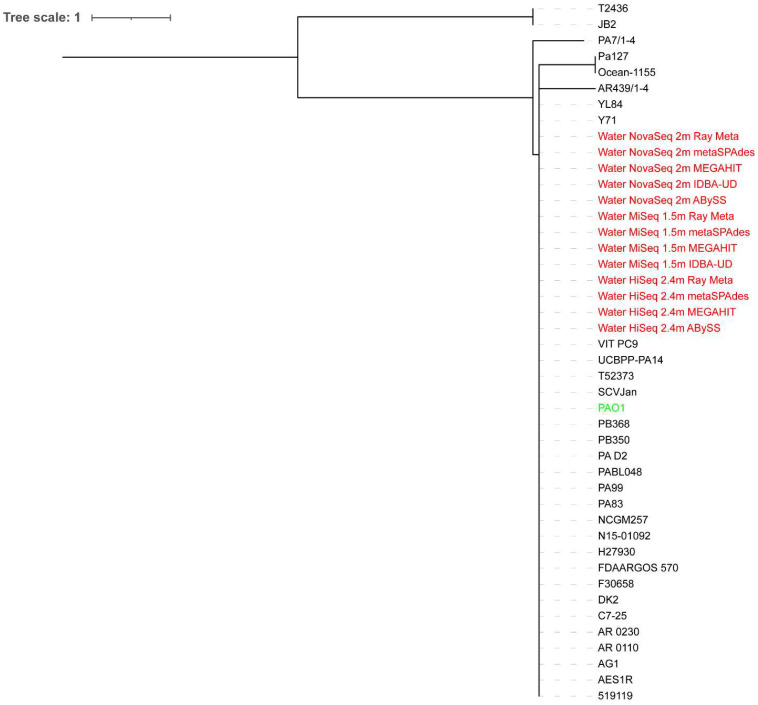
Whole-genome phylogenetic tree of extracted reads classified as *Pseudomonas* from the surface water metagenome assemblies of 2.4 million HiSeq, 1.5 million MiSeq, and 2 million NovaSeq reads in addition to *P. aeruginosa* PAO1 compared to 30 *P. aeruginosa* strains. The scale bar indicates the genetic distance. Assemblies are indicated in red, while *P. aeruginosa* PAO1 is indicated in green.

**Figure 4:**
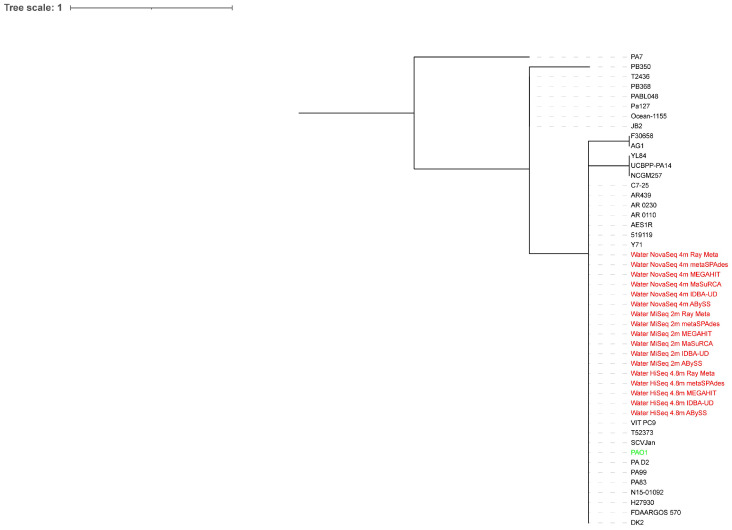
Whole-genome phylogenetic tree of extracted reads classified as *Pseudomonas* from the surface water metagenome assemblies of 4.8 million HiSeq, 2 million MiSeq, and 4 million NovaSeq reads in addition to *P. aeruginosa* PAO1 compared to 30 *P. aeruginosa* strains. The scale bar indicates the genetic distance. Assemblies are indicated in red, while *P. aeruginosa* PAO1 is indicated in green.

All extracted reads classified as *Pseudomonas* from the surface water metagenome assemblies were on a major clade where *P. aeruginosa* PAO1 and some *P. aeruginosa* strains were located, regardless of the sequencing depth (Figure 3 and Figure 4). The HiSeq assemblies were closer to *P. aeruginosa* PAO1 on the phylogenetic trees than the MiSeq and NovaSeq assemblies, with the ABySS assemblies of HiSeq reads performing the best at both sequencing depths.

## 4. Discussion

Given that benchmarking a metagenome assembler should not be governed by assembly quality statistics alone, in the current study, a strong emphasis has been laid on the depth of the metagenomic and genomic information of foodborne and waterborne pathogens that could be gained from the assembly for downstream analyses. Our study will help researchers understand the advantages and disadvantages of each assembler when identifying bacterial pathogens in a metagenomic context, thus facilitating the selection of the assembler best suited for achieving their research goals. Generally, the best assembly is performed by multi-*k*-mer assemblers such as IDBA-UD, MEGHIT, and metaSPAdes. These observations are in accordance with previous results by van der Walt et al. [41], who found that IDBA-UD, MEGHIT, and metaSPAdes are the best assemblers for environmental metagenomic datasets, especially for genome-centric studies.

Assemblers using a range of *k*-mers, including IDBA-UD, MEGHIT, and metaSPAdes, which maximize the read information that can be incorporated into the assembly, outperformed single *k*-mer assemblers, including ABySS, MaSuRCA, and Ray Meta. While the latter reconstructed only low-abundance metagenomes with small *k*-mers or high-abundance ones with large *k*-mers, using multi-*k*-mers could considerably enhance the recovered genome fraction and serve as a large driver of producing high-quality assemblies [42]. The choice of *k*-mer is critical when using single *k*-mer de Bruijn graph assemblers such as ABySS, MaSuRCA, and Ray Meta. Although small *k*-mers are more effective in building overlaps, they could collapse more repeats together and fail to resolve them, thus resulting in the de Bruijn graphs becoming more tangled [11]. Large *k*-mers typically generate longer contigs but may miss overlaps and are more susceptible to sequencing errors, particularly in low-coverage regions that are typical for metagenomic datasets. Most current metagenome assemblers, therefore, generate contigs from the de Bruijn graphs using multi-*k*-mers. Our results indicate that although IDBA-UD, MEGHIT, and metaSPAdes performed comparably, MEGAHIT emerged as the most memory-efficient assembler, which produced some of the best assemblies throughout this study while only using a fraction of the computational resources required by other assemblers. We, therefore, strongly recommend MEGAHIT for those who do not have access to sufficient computational resources.

A closer examination of the extracted reads classified as the pathogens of interest reveals that one major shortcoming is to yield accurate results for downstream genomic analyses, which may require further improvements in the assembly algorithms for satisfactory performance. Additionally, we suspect that the classification algorithm, reference taxonomies, and standardized database of Kraken 2 could also contribute to how metagenomic reads were classified and extracted in this research, thus casting doubt on the effectiveness of Kraken 2 on read extraction from metagenome assemblies. The accuracy and reliability of our genomic analyses using the extracted reads relied critically on the pre-built *k*-mer database of Kraken 2 and on the taxa that can be rapidly queried for exact matches to *k*-mers found in each metagenome assembly. As databases of assembled genomes continue to grow, databases of reference sequences used for aligning reads and mapping *k*-mers in metagenome assemblies will also mature [26].

While extracting whole-genomic data from the assembly for accurate genomic analyses remains challenging, our study illustrates that current assembly algorithms have resulted in reduced bias and improved resolution for whole-genome phylogeny. We, thus, demonstrate the potential of using the classified reads from metagenome assemblies for accurate phylogenetic inference, as revealed by the congruent phylogenetic topology between the reference genome and the assemblies. Still, care should be taken to interpret these results, especially when closely related strains (e.g., *Salmonella* strains with the same serotype) are aligned. In a routine clinical context, we do not suggest here that phylogenetic analysis can as yet be performed solely using the classified reads from metagenome assemblies, though we believe that information from these assemblies will complement other identification methods. We identified some recurrent phylogenetic patterns of metagenome assemblies of Illumina short reads that could potentially be addressed in the future. It is, therefore, anticipated that continued improvements to short-read assembly algorithms could systematically improve assembly quality to the point that an accurate phylogenetic inference could be achieved with the classified reads from metagenome assemblies. Until then, it is still necessary to use pure isolates from metagenomic samples to carry out an accurate phylogenetic analysis.

We also sought to determine how three different sequencing platforms, HiSeq, Missed, and NovaSeq, differ in the metagenomic identification of foodborne and waterborne pathogens. Unsurprisingly, we did note some platform-specific variations in our data. Meanwhile, we found that neither platform appeared to have a significant advantage regarding the metagenomic identification of foodborne and waterborne pathogens in most cases if the sequencing depth was held constant, though there were some noteworthy differences. Although MiSeq generally produced assemblies with higher quality compared to HiSeq and NovaSeq, HiSeq and NovaSeq outperformed MiSeq for AMR profiling at the same sequencing depth. When Frey et al. [43] compared Roche-454, Ion Torrent PGM, and MiSeq for the metagenomic identification of *Bacillus anthracis*, Dengue virus Type 1 and Type 2, and Influenza A virus in whole human blood, no one sequencing platform surpassed others in terms of all metagenomic and genomic analyses, which mirror the results presented here.

No universal standard has been developed to recommend what sequencing depth is needed to make an accurate metagenomic identification. There is also a paucity of knowledge regarding the actual sequencing depth needed for each sequencing platform. Obtaining sufficient sequencing depth for a complex metagenomic sample can normally yield a high volume of data. As expected, an increase in the sequencing depth permitted a more accurate metagenomic identification of *S.* Indiana SI43 and *P. aeruginosa* PAO1 in most cases. However, excessive coverage may also introduce more sequencing errors [8]. In support of this notion, our study reveals that *cmx* in *M. luteus* AS2 was present in the MEGAHIT and metaSPAdes assemblies of 1 million MiSeq reads, whereas the assemblies of 2.4 million HiSeq, 1.5 million MiSeq, and 2 million NovaSeq reads did not harbor *cmx*. Apart from the identification of ARGs, an increase in the sequencing depth of HiSeq and NovaSeq also led to inaccurate numbers of virulence genes of *S.* Indiana SI43. Meanwhile, the extracted reads classified as *Salmonella* from the ABySS, MEGAHIT, and Ray Meta assemblies of 1 million MiSeq reads were unambiguously serotyped. In contrast, when the sequencing depth of MiSeq was increased to 1.5 million reads, Ray Meta produced the only assembly with accurate serotyping.

We chose to benchmark the assemblers with their default parameters and recommended settings. Future optimization of these parameters and settings before implementation could potentially improve their assembly algorithms, allowing for a much more accurate reconstruction of bacterial communities. Finally, we must note that although benchmarking of assemblers such as this study are informative snapshots of performance, re-evaluation is warranted as assembly algorithms evolve rapidly. Considering our observations, we also urge researchers to carefully consider the assembler used while bearing in mind their research questions and the features of the metagenomic samples. Meanwhile, we acknowledge that the comparison of various next-generation sequencing technologies such as Illumina, PacBio, and Oxford Nanopore for the metagenomic identification of bacterial pathogens is also a significant area that requires future dedicated studies to establish the most appropriate algorithmic approaches for accurate results.

## 5. Conclusions

The main findings of our study on simulated bacterial communities indicate that overall, IDBA-UD, MEGHIT, and metaSPAdes outperformed (not in all aspects) other assemblers, whose superiority is most likely attributed to their multi-*k*-mer approaches. This work has highlighted the suitability of using these assemblers alone for metagenome assembly and paved the way toward the standardization of bioinformatic pipelines for assembling Illumina short reads. Our study presents the first critical assessment of metagenome assemblers of Illumina short reads derived from complex food and water samples for the metagenomic identification of bacterial pathogens. In sum, the present study supports metagenome assembly as a valuable technique for boosting contiguity and increasing the accuracy of pathogen identification but also emphasizes the choice of the right sequencing depth and platform to harness the full potential of the assembler. Our findings, thus, provide key information towards establishing a framework for guiding the selection of assembler and sequencing platform and depth, which will also help researchers to answer their specific microbiological questions.

## Figures and Tables

**Table 1:** Quality of the spinach metagenome assemblies.

Sequencing Depth (Million)	Sequencer	Assembler	Number of Contigs	Length of the Largest Contig (bp)	Total Length (bp)	N50	L50	Complete BUSCOs (%)	Fragmented BUSCOs (%)	Missing BUSCOs (%)
1	HiSeq	ABySS	2547	2816	583,551	906	7	N.A. ^a^	N.A.	N.A.
		IDBA-UD	19,390	30,957	18,981,326	2446	1327	85.5	12.9	1.6
		MaSuRCA	4169	4169	4,250,085	1877	601	12.1	37.9	50.0
		MEGAHIT	22,873	15,323	20,016,585	1508	3034	83.8	14.5	1.7
		metaSPAdes	45,708	46,199	30,661,187	3887	1130	86.3	12.1	1.6
		Ray Meta	91,979	2679	19,109,345	660	1493	25.0	58.9	16.1
	MiSeq	ABySS	38,624	131,924	29,026,490	8322	453	96.8	3.2	0.0
		IDBA-UD	38,905	605,513	48,155,638	16,200	329	100.0	0.0	0.0
		MaSuRCA	6317	6317	25,421,869	41,463	68	99.2	0.8	0.0
		MEGAHIT	69,714	167,091	62,018,965	2525	3241	100.0	0.0	0.0
		metaSPAdes	39,536	484,133	56,431,406	7826	700	100.0	0.0	0.0
		Ray Meta	107,349	8316	34,485,996	1052	3854	72.6	24.2	3.2
	NovaSeq	ABySS	23,285	3465	6,799,130	613	584	N.A.	N.A.	N.A.
		IDBA-UD	19,309	102,980	24,348,504	6950	519	96.0	4.0	0.0
		MaSuRCA	4915	4915	9,411,060	5142	517	N.A.	N.A.	N.A.
		MEGAHIT	25,109	26,002	26,423,629	2219	2325	98.4	0.8	0.8
		metaSPAdes	53,521	144,898	38,848,690	5595	745	100.0	0.0	0.0
		Ray Meta	91,241	3513	23,595,378	775	2849	N.A.	N.A.	N.A.
2.4	HiSeq	ABySS	96,154	399,012	38,151,970	20,826	119	100.0	0.0	0.0
		IDBA-UD	20,986	982,298	36,504,325	33,886	121	100.0	0.0	0.0
		MaSuRCA	3792	1,157,404	22,062,604	127,766	26	100.0	0.0	0.0
		MEGAHIT	25,891	415,309	38,209,837	6671	623	100.0	0.0	0.0
		metaSPAdes	136,810	18,480	39,795,754	1305	3669	100.0	0.0	0.0
		Ray Meta	54,095	701,901	51,025,031	26,733	200	77.4	21.0	1.6
2	NovaSeq	ABySS	76,438	239,683	35,384,363	13,564	229	100.0	0.0	0.0
		IDBA-UD	22,191	866,390	38,814,884	43,320	106	100.0	0.0	0.0
		MaSuRCA	4662	1,341,366	23,872,171	107,965	33	100.0	0.0	0.0
		MEGAHIT	28,959	333,683	41,721,455	6365	742	100.0	0.0	0.0
		metaSPAdes	129,147	14,900	40,184,250	1286	3904	100.0	0.0	0.0
		Ray Meta	61,622	788,082	55,891,970	22,000	211	79.8	16.1	4.1
1.5	MiSeq	ABySS	27,450	985,657	31,190,322	23,037	88	100.0	0.0	0.0
		IDBA-UD	46,339	830,885	59,634,527	26,442	222	100.0	0.0	0.0
		MaSuRCA	7482	1,649,401	35,047,685	142,499	35	100.0	0.0	0.0
		MEGAHIT	72,884	396,248	72,945,424	3241	2413	100.0	0.0	0.0
		metaSPAdes	41,606	1,238,639	67,167,714	11,584	489	100.0	0.0	0.0
		Ray Meta	133,221	174,311	43,254,966	10,098	355	98.4	1.6	0.0

^a^ N.A., not applicable due to errors produced by BUSCO.

**Table 2:** Quality of the surface water metagenome assemblies.

Sequencing Depth (Million)	Sequencer	Assembler	Number of Contigs	Length of the Largest Contig (bp)	Total Length (bp)	N50	L50	Complete BUSCOs (%)	Fragmented BUSCOs (%)	Missing BUSCOs (%)
2.4	HiSeq	ABySS	94,776	567,651	38,437,605	29,385	156	100.0	0.0	0.0
		IDBA-UD	27,858	675,083	38,978,604	42,320	125	100.0	0.0	0.0
		MaSuRCA	8722	1,065,770	31,493,699	144,020	49	100.0	0.0	0.0
		MEGAHIT	31,551	506,253	40,500,481	8125	595	100.0	0.0	0.0
		metaSPAdes	49,253	598,239	51,906,012	6280	480	100.0	0.0	0.0
		Ray Meta	129,379	40,462	40,407,420	2512	2071	98.3	1.6	0.1
1.5	MiSeq	ABySS	27,664	1,156,931	31,438,774	66,328	81	100.0	0.0	0.0
		IDBA-UD	45,943	694,199	62,390,072	13,321	525	100.0	0.0	0.0
		MaSuRCA	10,851	1,084,592	40,632,725	74,717	82	100.0	0.0	0.0
		MEGAHIT	53,243	506,136	66,833,415	3602	2396	100.0	0.0	0.0
		metaSPAdes	26,622	598,615	62,354,405	10,634	818	100.0	0.0	0.0
		Ray Meta	128,576	55,589	42,575,322	4620	1054	99.2	0.8	0.0
2	NovaSeq	ABySS	75,321	294,921	35,485,842	15,726	253	100.0	0.0	0.0
		IDBA-UD	28,508	1,067,518	41,475,688	31,641	144	100.0	0.0	0.0
		MaSuRCA	9695	826,913	32,976,162	107,348	61	100.0	0.0	0.0
		MEGAHIT	33,868	348,830	44,148,453	5785	692	100.0	0.0	0.0
		metaSPAdes	49,929	595,582	54,925,053	6036	613	100.0	0.0	0.0
		Ray Meta	118,667	24,869	40,595,237	2461	2217	96.8	3.2	0.0
4.8	HiSeq	ABySS	68,676	860,765	49,469,839	49,695	112	100.0	0.0	0.0
		IDBA-UD	22,240	986,994	55,895,382	21,745	345	100.0	0.0	0.0
		MaSuRCA	N.A. ^a^	N.A.	N.A.	N.A.	N.A.	100.0	0.0	0.0
		MEGAHIT	27,805	582,024	57,905,030	7080	925	100.0	0.0	0.0
		metaSPAdes	27,267	1,010,681	63,473,426	30,990	354	100.0	0.0	0.0
		Ray Meta	129,559	69,858	55,853,404	2606	1961	100.0	0.0	0.0
2	MiSeq	ABySS	34,265	1,086,290	38,581,270	99,221	62	100.0	0.0	0.0
		IDBA-UD	48,934	598,213	69,472,563	27,024	385	100.0	0.0	0.0
		MaSuRCA	9240	1,692,548	49,934,547	35,328	177	100.0	0.0	0.0
		MEGAHIT	46,433	598,268	70,400,115	5687	1701	100.0	0.0	0.0
		metaSPAdes	19,644	1,345,803	65,375,463	25,509	452	100.0	0.0	0.0
		Ray Meta	138,343	93,582	49,496,982	5951	783	100.0	0.0	0.0
4	NovaSeq	ABySS	81,690	859,123	53,258,171	51,140	115	100.0	0.0	0.0
		IDBA-UD	20,992	1,080,025	57,665,814	32,176	276	100.0	0.0	0.0
		MaSuRCA	6872	741,283	56,276,212	87,439	151	100.0	0.0	0.0
		MEGAHIT	27,425	599,130	60,289,818	8523	895	100.0	0.0	0.0
		metaSPAdes	25,754	1,080,366	64,824,870	38,468	320	100.0	0.0	0.0
		Ray Meta	118,206	64,911	56,050,840	2454	2283	99.2	0.8	0.0

^a^ N.A., not applicable due to errors produced by BUSCO.

**Table 3:** Plasmids in the spinach metagenome assemblies.

Sequencing Depth (Million)	Sequencer	Assembler						Reference
		ABySS	IDBA-UD	MaSuRCA	MEGAHIT	metaSPAdes	Ray Meta	
1	HiSeq	N.D. ^a^	IncHI2A IncHI2 IncQ1	IncHI2	IncHI2A IncHI2 IncQ1	IncHI2A IncHI2 IncQ1	IncHI2	IncHI2A IncHI2 IncQ1
	MiSeq	IncHI2A IncHI2 IncQ1	IncHI2A IncHI2 IncQ1	IncHI2A IncHI2 IncQ1 IncQ1 IncQ1	IncHI2A IncHI2 IncQ1	IncHI2A IncHI2 IncQ1	IncHI2 IncQ1	
	NovaSeq	IncHI2	IncHI2A IncHI2 IncQ1	IncHI2A IncHI2 IncQ1	IncHI2A IncHI2 IncQ1	IncHI2A IncHI2 IncQ1	IncHI2 IncQ1	
2.4	HiSeq	IncHI2A IncHI2 IncQ1	IncHI2A IncHI2 IncQ1	IncHI2A IncHI2 IncQ1	IncHI2A IncHI2 IncQ1	IncHI2A IncHI2 IncQ1	IncHI2A IncHI2 IncQ1	
2	NovaSeq	IncHI2A	IncHI2A IncHI2 IncQ1	IncHI2A IncQ1	IncHI2A IncHI2 IncQ1	IncHI2A IncHI2 IncQ1	IncHI2A	
1.5	MiSeq	IncHI2 IncHI2A IncQ1	IncHI2 IncHI2A IncQ1	IncHI2 IncHI2A IncQ1	IncHI2 IncHI2A IncQ1	IncHI2 IncHI2A IncQ1	IncHI2 IncHI2A IncQ1	

^a^ N.D., not detectable.

**Table 4:** Numbers of virulence genes in the spinach metagenome assemblies.

Sequencing Depth (Million)	Sequencer	Assembler						Reference
		ABySS	IDBA-UD	MaSuRCA	MEGAHIT	metaSPAdes	Ray Meta	
1	HiSeq	0	66	15	69	79	20	91
	MiSeq	87	91	90	91	91	55	
	NovaSeq	10	90	37	90	90	39	
2.4	HiSeq	112	122	105	123	126	100	
2	NovaSeq	111	124	105	124	126	97	
1.5	MiSeq	90	93	91	91	89	91	

**Table 5:** Numbers of virulence genes in the surface water metagenome assemblies.

Sequencing Depth (Million)	Sequencer	Assembler						Reference
		ABySS	IDBA-UD	MaSuRCA	MEGAHIT	metaSPAdes	Ray Meta	
2.4	HiSeq	1	3	1	6	19	0	241
1.5	MiSeq	1	65	3	104	94	7	
2	NovaSeq	0	6	1	11	25	1	
4.8	HiSeq	8	54	N.A. ^a^	69	93	12	
2	MiSeq	4	114	15	151	147	10	
4	NovaSeq	16	68	19	88	106	12	

^a^ N.A., not applicable.

**Table 6:** *Salmonella* pathogenicity island (SPI) typing of the spinach metagenome assemblies.

Sequencing Depth (Million)	Sequencer	Assembler						Reference
		ABySS	IDBA-UD	MaSuRCA	MEGAHIT	metaSPAdes	Ray Meta	
1	HiSeq	N.D. ^a^	SPI-1 (7) SPI-2 (7) SPI-3 (2)	SPI-2 (3)	SPI-1 (6) SPI-2 (7) SPI-3 (2)	SPI-1 (8) SPI-2 (7) SPI-3 (2)	SPI-2 (2)	SPI-1 (8) SPI-2 (6) SPI-3 (3) SPI-4 (1) SPI-5 (1) SPI-9 (1)
	MiSeq	C63PI (1) SPI-1 (5) SPI-2 (8) SPI-3 (2) SPI-4 (1)	SPI-1 (8) SPI-2 (6) SPI-3 (2) SPI-4 (1) SPI-5 (1) SPI-9 (1)	C63PI (1) SPI-1 (7) SPI-2 (8) SPI-3 (2)	C63PI (1) SPI-1 (7) SPI-2 (6) SPI-3 (2) SPI-5 (1)	SPI-1 (8) SPI-2 (6) SPI-3 (3) SPI-5 (1) SPI-9 (1)	SPI-1 (3) SPI-2 (5) SPI-3 (1)	
	NovaSeq	N.D	C63PI (1) SPI-1 (5) SPI-2 (7) SPI-3 (2)	SPI-1 (1) SPI-2 (2)	C63PI (1) SPI-1 (5) SPI-2 (7) SPI-3 (2)	C63PI (1) SPI-1 (7) SPI-2 (5) SPI-3 (3) SPI-5 (1) SPI-9 (1)	SPI-1 (3) SPI-2 (2) SPI-3 (1)	
2.4	HiSeq	SPI-1 (8) SPI-2 (6) SPI-3 (3) SPI-5 (1) SPI-9 (1)	SPI-1 (8) SPI-2 (6) SPI-3 (2) SPI-4 (1) SPI-5 (1) SPI-9 (1)	SPI-1 (8) SPI-2 (9) SPI-3 (2) SPI-4 (1) SPI-5 (1) SPI-9 (1)	SPI-1 (8) SPI-2 (6) SPI-3 (2) SPI-5 (1) SPI-9 (1)	SPI-1 (8) SPI-2 (6) SPI-3 (3) SPI-4 (1) SPI-5 (1) SPI-9 (1)	SPI-1 (8) SPI-2 (8) SPI-3 (2)	
2	NovaSeq	SPI-1 (8) SPI-2 (8) SPI-3 (2)	SPI-1 (8) SPI-2 (6) SPI-3 (2) SPI-4 (1) SPI-5 (1) SPI-9 (1)	SPI-1 (7) SPI-2 (7) SPI-3 (2) SPI-5 (1) SPI-9 (1)	SPI-1 (8) SPI-2 (6) SPI-3 (2) SPI-5 (1) SPI-9 (1)	SPI-1 (8) SPI-2 (6) SPI-3 (3) SPI-4 (1) SPI-5 (1) SPI-9 (1)	SPI-1 (8) SPI-2 (6) SPI-3 (2)	
1.5	MiSeq	SPI-1 (8) SPI-2 (6) SPI-3 (3) SPI-4 (1) SPI-5 (1) SPI-9 (1)	SPI-1 (8) SPI-2 (6) SPI-3 (3) SPI-5 (1) SPI-9 (1)	SPI-1 (8) SPI-2 (6) SPI-3 (3) SPI-4 (1) SPI-5 (1) SPI-9 (1)	SPI-1 (8) SPI-2 (6) SPI-3 (2) SPI-5 (1) SPI-9 (1)	SPI-1 (8) SPI-2 (6) SPI-3 (3) SPI-4 (1) SPI-5 (1) SPI-9 (1)	SPI-1 (8) SPI-2 (4) SPI-3 (3) SPI-5 (1) SPI-9 (1)	

^a^ N.D., not detectable.

## Data Availability

Not applicable.

## Supplementary Materials

The following supporting information can be downloaded at: https://www.mdpi.com/article/10.3390/microorganisms10122416/s1, Table S1: Relative abundance of each phylum in the simulated bacterial community on spinach; Table S2: Relative abundance of each phylum in the simulated bacterial community in surface water; Table S3: Quality of the reads classified as *Salmonella* in the spinach metagenome assemblies; Table S4: Quality of the surface water metagenome assemblies; Table S5: Antimicrobial resistance genotypes of the spinach metagenome assemblies with 1 million reads; Table S6: Additional antimicrobial resistance genes detected and those identified not to be in the reference genomes; Table S7: Predicted antimicrobial resistance phenotypes of the spinach metagenome assemblies; Table S8: Antimicrobial resistance genotypes of the spinach metagenome assemblies with 1.5 million MiSeq reads; Table S9: Additional antimicrobial resistance genes detected and those identified not to be in the reference genomes; Table S10: Predicted antimicrobial resistance phenotypes of the spinach metagenome assemblies with 1.5 million MiSeq reads; Table S11: Antimicrobial resistance genotypes of the spinach metagenome assemblies with 2.4 million HiSeq reads and 2 million NovaSeq reads; Table S12: Additional antimicrobial resistance genes detected and those identified not to be in the reference genomes; Table S13: Predicted antimicrobial resistance phenotypes of the spinach metagenome assemblies with 2.4 million HiSeq reads and 2 million NovaSeq reads; Table S14: Antimicrobial resistance genotypes of the surface water metagenome assemblies with 1.5 million MiSeq reads; Table S15: Predicted antimicrobial resistance phenotypes of the spinach metagenome assemblies with 1.5 million MiSeq reads; Table S16: Antimicrobial resistance genotypes of the surface water metagenome assemblies with 2.4 million HiSeq reads and two million NovaSeq reads; Table S17: Predicted antimicrobial resistance phenotypes of the surface water metagenome assemblies with 2.4 million HiSeq reads and two million NovaSeq reads; Table S18: Antimicrobial resistance genotypes of the surface water metagenome assemblies with 4.8 million HiSeq reads, two million MiSeq reads, and four million NovaSeq reads; Table S19: Predicted antimicrobial resistance phenotypes of the surface water metagenome assemblies with 4.8 million HiSeq reads, two million MiSeq reads, and four million NovaSeq reads; Table S20: Antimicrobial resistance genotypes of the reads classified as *Salmonella* in the spinach metagenome assemblies with one million reads; Table S21: Predicted antimicrobial resistance phenotypes of the reads classified as *Salmonella* in the spinach metagenome assemblies with one million reads; Table S22: Antimicrobial resistance genotypes of the reads classified as *Salmonella* in the spinach metagenome assemblies with 1.5 million MiSeq reads; Table S23: Predicted antimicrobial resistance phenotypes of the reads classified as *Salmonella* in the spinach metagenome assemblies with 1.5 million MiSeq reads; Table S24: Antimicrobial resistance genotypes of the reads classified as *Salmonella* in the spinach metagenome assemblies with 2.4 million HiSeq reads and 2 million NovaSeq reads; Table S25: Predicted antimicrobial resistance phenotypes of the reads classified as *Salmonella* in the spinach metagenome assemblies with 2.4 million HiSeq reads and 2 million NovaSeq reads; Table S26: Antimicrobial resistance genotypes of the reads classified as *Pseudomonas* in the surface water metagenome assemblies with 1.5 million MiSeq reads; Table S27: Antimicrobial resistance genotypes of the reads classified as *Pseudomonas* in the surface water metagenome assemblies with 2.4 million HiSeq reads and 2 million NovaSeq reads; Table S28: Predicted antimicrobial resistance phenotypes of the reads classified as *Pseudomonas* in the surface water metagenome assemblies with 2.4 million HiSeq reads and 2 million NovaSeq reads; Table S29: Antimicrobial resistance genotypes of the reads classified as *Pseudomonas* in the surface water metagenome assemblies with 4.8 million HiSeq reads, two million MiSeq reads, and four million NovaSeq reads; Table S30: Predicted antimicrobial resistance phenotypes of the reads classified as *Pseudomonas* in the surface water metagenome assemblies with 4.8 million HiSeq reads, two million MiSeq reads, and four million NovaSeq reads; Table S31: Numbers of virulence genes in the reads classified as *Salmonella* in the spinach metagenome assemblies; Table S32: Numbers of virulence genes in the reads classified as *Pseudomonas* in the surface water metagenome assemblies; Table S33: *Salmonella* pathogenicity island (SPI) typing of the reads classified as *Salmonella* in the spinach metagenome assemblies; Table S34: *Salmonella* serotyping of the reads classified as *Salmonella* in the spinach metagenome assemblies; Table S35: Multilocus sequence typing (MLST) of the reads classified as *Salmonella* in the spinach metagenome assemblies.
