# Supplementary material for: Critical Assessment of Short-Read Assemblers for the Metagenomic Identification of Foodborne and Waterborne Pathogens Using Simulated Bacterial Communities
Source: Microorganisms. 2022 Dec 6;10(12):2416. doi: 10.3390/microorganisms10122416 (PMC9784204; doi:10.3390/microorganisms10122416)
Supplement: Supplementary file 1 [file microorganisms-10-02416-s001.zip › microorganisms-2056135-supplementary.pdf]

Table S1 Relative abundance of each phylum in the simulated bacterial community on spinach

Phylum	Family and/or genus	Relative abundance (%)	Representative microorganism	RefSeq assembly accession	GenBank assembly accession
Actinobacteria	Corynebacterineae	0.41	*Rhodococcus biphenylivorans* TG9	GCF_003288095.1	GCA_ 003288095.1
	Propionibacterineae	2.61	*Propionibacterium freudenreichii* FAM 14217	GCF_013205725.1	GCA_ 013205725.1
	Micrococcineae	1.40	*Micrococcus luteus* AS2	GCF_005280335.1	GCA_ 005280335.1
Acidobacteria	Gp4	2.03	*Chloracidobacterium* sp. N	GCF_018304765.1	GCA_ 018304765.1
	Gp6	2.66	*Luteitalea pratensis* DSM 100886	GCF_001618865.1	GCA_ 001618865.1
Deinococcus-Thermus	*Deinococcus* spp.	2.97	*Deinococcus ruber* JCM 31311	GCF_014648095.1	GCA_ 014648095.1
Firmicutes	*Exiguobacterium* spp.	0.05	*Exiguobacterium acetylicum* AMCC 101217	GCF_008274845.1	GCA_ 008274845.1
Alpha-proteobacteria	Sphingomonadaceae	2.39	*Sphingomonas alpina* DSM 22537	GCF_009720245.1	GCA_ 009720245.1
	Sphingomonadales	1.08	*Sphingomonas alpina* DSM 22537	GCF_009720245.1	GCA_ 009720245.1
	Rhizobiales	1.22	*Bradyrhizobium arachidis* CCBAU 051107	GCF_015291705.1	GCA_ 015291705.1
	Unclassified alpha-proteobacteria	1.04	*Brevundimonas diminuta* FDAARGOS 1026	GCF_016127655.1	GCA_ 016127655.1
	Sphingomonas spp.	6.44	*Sphingomonas alpina* DSM 22537	GCF_009720245.1	GCA_ 009720245.1
	Methylobacterium spp.	1.22	*Methylobacterium oryzae* CBMB20	GCF_000757795.1	GCA_ 000757795.1
	Rhizobium spp.	1.22	*Rhizobium* sp. S41	GCF_001691455.1	GCA_ 001691455.1
	Brevundimonas	2.79	*Brevundimonas diminuta* FDAARGOS 1026	GCF_016127655.1	GCA_ 016127655.1
Gamma-proteobacteria	Pseudomonadaceae	0.41	*Pseudomonas fluorescens* NCTC10038	GCF_900475215.1	GCA_ 900475215.1
	Xanthomonadaceae	1.40	Xanthomonas oryzae pv. oryzicola YM15	GCF_001021915.1	GCA_ 001021915.1
	Acinetobacter spp.	1.53	Acinetobacter oleivorans DR1	GCF_000196795.1	GCA_ 000196795.1
	Pseudomonas spp.	9.19	Pseudomonas fluorescens NCTC10038	GCF_900475215.1	GCA_ 900475215.1
	Stenotrophomonas spp .	0.32	Stenotrophomonas maltophilia NCTC10258	GCF_900475405.1	GCA_ 900475405.1
	Pantoea spp.	0.14	Pantoea ananatis LCFJ-001	GCF_016598655.1	GCA_ 016598655.1
	Comamonadaceae	2.30	Diaphorobacter ruginosibacter DSM 27467	GCF_014395975.1	GCA_ 014395975.1

Beta-proteobacteria	Oxalobacteraceae	8.55	Massilia flava DSM 26639	GCF_009789595.1	GCA_ 009789595.1
	Burkholderiales	1.26	Rhizobacter gummiphilus NBRC 109400	GCF_002762215.1	GCA_ 002762215.1
	Unclassified beta-proteobacteria	0.95	Massilia flava DSM 26639	GCF_009789595.1	GCA_ 009789595.1
	Ralstonia spp.	1.22	Ralstonia pickettii FDAARGOS 410	GCF_002393485.1	GCA_ 002393485.1
	Naxibacter spp.	3.74	Massilia flava DSM 26639	GCF_009789595.1	GCA_ 009789595.1
	Massilia spp.	7.38	Massilia flava DSM 26639	GCF_009789595.1	GCA_ 009789595.1

Table S2 Relative abundance of each phylum in the simulated bacterial community in surface water

Phylum	Family and/or genus	Relative abundance (%)	Representative microorganism	RefSeq assembly accession	GenBank assembly accession
Actinobacteria	Actinomycetales	6	*Saccharopolyspora erythraea* NRRL 2338	GCF_000062885.1	GCA_000062885.1
	Acidimicrobiales	1	*Acidimicrobium ferrooxidans* DSM 10331	GCF_000023265.1	GCA_000023265.1
Acidobacteria	Sphingobacteriales	10	*Sphingobacterium psychroaquaticum* SJ-25	GCF_004421025.1	GCA_004421025.1
Alpha-proteobacteria	Rhodospirillales	7	*Magnetospirillum gryphiswaldense* MSR-1	GCF_000513295.1	GCA_000513295.1
	Rhodobacterales	1	*Pikeienuella piscinae* RR4-56	GCF_011044155.1	GCA_011044155.1
	Rhizobiales	1	*Blastochloris viridis* ATCC 19567	GCF_001402875.1	GCA_001402875.1
Gamma-proteobacteria	Alteromonadales	11	*Alteromonas naphthalenivorans* SN2	GCF_000213655.1	GCA_000213655.1
	Pseudomonadales	2	*Pseudomonas aeruginosa* PAO1	GCF_000006765.1	GCA_000006765.1
	Aeromonadales	11	*Tolumonas auensis* DSM 9187	GCF_000023065.1	GCA_000023065.1
	Xanthomonadales	2	*Lysobacter caseinilyticus* KVB24	GCF_018406605.1	GCA_018406605.1
Beta-proteobacteria	Burkholderiales	4	*Pandoraea norimbergensis* DSM 11628	GCF_001465545.3	GCA_001465545.3
Cyanobacteria	Cyanobacteriaceae	12	*Cyanobacterium aponinum* PCC 10605	GCF_000317675.1	GCA_000317675.1
Bacteroidetes	Flavobacteriales	3	*Gramella flava* JLT2011	GCF_001951155.1	GCA_001951155.1
Planctomycetes	Planctomycetales	2	*Planctopirus ephydatiae* spb1	GCF_007752345.1	GCA_007752345.1
Verrucomicrobia	Puniceicoccales	3	*Coraliomargarita akajimensis* DSM 45221	GCF_000025905.1	GCA_000025905.1

Table S3 Quality of the reads classified as *Salmonella* in the spinach metagenome assemblies

Sequencing depth (million)	Sequencer	Assembler	Number of contigs	Length of the largest contig (bp)	Total length (bp)	GC content (%)	N50	L50	Number of misassemblies	Genome fraction (%)	Number of N's per 100 kbp	Number of mismatches per 100 kbp	Number of indels per 100 kbp
1	HiSeq	ABySS	888	2,032	205,270	54.62	875	3	0	0.17	0	0	0
		IDBA-UD	1,505	13,939	1,601,914	53.11	1,784	183	18	28.01	29.73	44.97	1.22
		MaSuRCA	1,131	6,745	1,051,894	52.76	1,541	185	10	15.56	10,807.09	7.08	26.89
		MEGAHIT	2,148	7,630	2,508,451	52.98	1,685	439	6	46.26	0	49.73	2.21
		metaSPAdes	753	13,831	928,597	52.79	3,943	63	3	15.69	2,093.10	60.23	25.78
		Ray Meta	13,484	2,679	3,615,154	52.77	655	479	0	16.00	0	0.38	0
	MiSeq	ABySS	482	19,967	487,069	52.35	5,296	24	1	7.73	844.63	11.92	6.22
		IDBA-UD	789	16,052	274,384	48.94	4,521	3	1	0.92	758.06	98.05	2.18
		MaSuRCA	6	3,852	7,810	48.74	3,852	1	0	0.14	2,875.27	41.77	0
		MEGAHIT	471	15,863	263,622	51.7	5,693	8	0	2.55	0	196.22	5.49
		metaSPAdes	50	8,078	18,878	52.8	8,078	1	0	0.24	0	84.20	0
		Ray Meta	6,138	6,842	3,149,115	52.87	1,124	598	0	44.54	0	2.70	0
	NovaSeq	ABySS	6,439	1,419	1,947,959	52.99	601	193	0	5.75	0	7.31	0
		IDBA-UD	336	15,272	383,150	51.91	3,104	25	3	6.77	57.34	65.04	1.48
		MaSuRCA	682	15,962	1,261,228	52.84	3,543	104	7	21.61	8,414.04	15.11	51.90
		MEGAHIT	882	16,255	1,638,975	52.87	3,103	162	3	31.39	0	51.87	1.60
		metaSPAdes	230	9,736	215,202	51.99	3,735	14	0	3.42	790.77	85	16.41
		Ray Meta	9,664	3,020	3,745,750	52.69	779	814	0	35.05	0	0.8	0.06
2.4	HiSeq	ABySS	253	8,306	89,439	51.63	3,526	4	1	0.79	965.74	27.79	2.53
		IDBA-UD	63	4,413	24,465	51.31	2,685	2	0	0.27	0	15	0
		MaSuRCA	7	9,236	17,028	53.03	9,236	1	0	0.32	0	37.44	31.2
		MEGAHIT	34	11,609	60,360	53.72	4,677	4	0	1.09	0	22.07	0
		metaSPAdes	33	13,784	32,959	52.81	4,472	2	0	0.57	0	49.45	0
		Ray Meta	1,476	9,282	2,123,339	53.37	2,385	270	0	39.53	0	0.51	0
2	NovaSeq	ABySS	382	8,815	202,085	52.46	1,293	16	0	2.60	187.01	12.32	2.31
		IDBA-UD	69	4,413	27,547	50.40	2,798	3	0	0.30	162.8	27.17	6.79
		MaSuRCA	22	6,984	23,023	51.60	3,620	2	1	0.37	421.62	120.35	27.35
		MEGAHIT	55	4,331	38,437	52.89	1,779	5	0	0.48	0	41.64	0
		metaSPAdes	44	4,472	21,781	53.97	1,981	3	0	0.30	0	0	0
		Ray Meta	1,719	9,129	2,314,038	53.20	2,253	304	0	42.33	0	0.9	0
1.5	MiSeq	ABySS	38	5,544	18,747	51.12	5,544	1	0	0.16	0	51.71	12.93
		IDBA-UD	1,446	4,413	450,935	53.25	776	10	2	0.66	0	111.60	0
		MaSuRCA	2	21,024	24,300	54.52	21,024	1	1	0.49	0	32.92	4.12
		MEGAHIT	134	13,925	80,859	53.80	4,609	3	0	0.89	0	122.17	0
		metaSPAdes	31	4,472	13,983	51.97	2,838	2	0	0.19	0	0	0
		Ray Meta	121	22,678	147,630	52.74	6,354	5	0	2.59	448.39	2.32	0

Table S4 Quality of the surface water metagenome assemblies

Sequencing depth (million)	Sequencer	Assembler	Number of contigs	Length of the largest contig (bp)	Total length (bp)	N50	L50
2.4	HiSeq	ABySS	94,776	567,651	38,437,605	29,385	156
		IDBA-UD	27,858	675,083	38,978,604	42,320	125
		MaSuRCA	8,722	1,065,770	31,493,699	144,020	49
		MEGAHIT	31,551	506,253	40,500,481	8,125	595
		metaSPAdes	49,253	598,239	51,906,012	6,280	480
		Ray Meta	129,379	40,462	40,407,420	2,512	2,071
1.5	MiSeq	ABySS	27,664	1,156,931	31,438,774	66,328	81
		IDBA-UD	45,943	694,199	62,390,072	13,321	525
		MaSuRCA	10,851	1,084,592	40,632,725	74,717	82
		MEGAHIT	53,243	506,136	66,833,415	3,602	2,396
		metaSPAdes	26,622	598,615	62,354,405	10,634	818
		Ray Meta	128,576	55,589	42,575,322	4,620	1,054
2	NovaSeq	ABySS	75,321	294,921	35,485,842	15,726	253
		IDBA-UD	28,508	1,067,518	41,475,688	31,641	144
		MaSuRCA	9,695	826,913	32,976,162	107,348	61
		MEGAHIT	33,868	348,830	44,148,453	5,785	692
		metaSPAdes	49,929	595,582	54,925,053	6,036	613
		Ray Meta	118,667	24,869	40,595,237	2,461	2,217
4.8	HiSeq	ABySS	68,676	860,765	49,469,839	49,695	112
		IDBA-UD	22,240	986,994	55,895,382	21,745	345
		MaSuRCA	N.A.[a]	N.A.	N.A.	N.A.	N.A.
		MEGAHIT	27,805	582,024	57,905,030	7,080	925
		metaSPAdes	27,267	1,010,681	63,473,426	30,990	354
		Ray Meta	129,559	69,858	55,853,404	2,606	1,961
2	MiSeq	ABySS	34,265	1,086,290	38,581,270	99,221	62
		IDBA-UD	48,934	598,213	69,472,563	27,024	385
		MaSuRCA	9,240	1,692,548	49,934,547	35,328	177
		MEGAHIT	46,433	598,268	70,400,115	5,687	1,701
		metaSPAdes	19,644	1,345,803	65,375,463	25,509	452
		Ray Meta	138,343	93,582	49,496,982	5,951	783
4	NovaSeq	ABySS	81,690	859,123	53,258,171	51,140	115
		IDBA-UD	20,992	1,080,025	57,665,814	32,176	276
		MaSuRCA	6,872	741,283	56,276,212	87,439	151
		MEGAHIT	27,425	599,130	60,289,818	8,523	895
		metaSPAdes	25,754	1,080,366	64,824,870	38,468	320
		Ray Meta	118,206	64,911	56,050,840	2,454	2,283

[a]N.A., not applicable.

Table S5 Antimicrobial resistance genotypes of the spinach metagenome assemblies with 1 million reads[a]

Microorganism	Antimicrobial resistance gene identified in the reference	Assembler[b]																	
		HiSeq						MiSeq						NovaSeq					
		A	I	C	H	S	R	A	I	C	H	S	R	A	I	C	H	S	R
M. luteus AS2	*cmx*																		
	sul1																		
Rhizobium sp. S41	*aph(6)-Id*																		
	strA																		
S. maltophilia NCTC10258	*aph(3')-IIc*																		
S. Indiana SI43	*ARR-3*																		
	aac(3)-IId																		
	aac(3)-IVa																		
	aac(6')-Ib-cr																		
	aac(6')-Ib-cr																		
	aadA2																		
	aph(3'')-Ib																		
	aph(4)-Ia																		
	aph(6)-Id																		
	blaCTX-M-65																		
	blaOXA-1																		
	blaTEM-1B																		
	catB4																		
	dfrA12																		
	floR																		
	fosA3																		
	rmtB																		
	sul1																		
	sul1																		
	sul2																		
	sul2																		

	tet(A)		■		■	■		■	■	■	■	■			■		■	■	
	gyrA (D87N)							■	■	■	■	■			■		■	■	
	gyrA (S83F)							■	■	■	■	■			■		■	■	
	parC (S80R)							■	■	■	■	■			■		■	■	

[a]Green block indicates that the antimicrobial resistance gene was detected and confirmed to be originated from its corresponding reference genome, while yellow block indicates that it was detected but not confirmed to be originated from its corresponding reference genome and white block indicates that the antimicrobial resistance gene was not detected. Yellow block with a cross indicates that *aph(6)-Id* or *sul1* was not confirmed to be originated from *S.* Indiana SI43 or other microorganisms.

[b]A, ABySS; I, IDBA-UD; C, MaSuRCA; H, MEGAHIT; S, metaSPAdes; R, Ray Meta.

Table S6 Additional antimicrobial resistance genes detected and those identified not to be in the reference genomes

Sequencer	Assembler	Antimicrobial resistance genes
HiSeq	MEGAHIT	*blaCTX-M-90*[a], *blaTEM-30*
	metaSPAdes	*blaTEM-30* (*S.* Indiana SI43)
	Ray Meta	*blaOXA-320*
MiSeq	ABySS	*sul1* (*S.* Indiana SI43)
	MaSuRCA	*sul2*

[a]The antimicrobial resistance gene with an underline was not present in the reference genomes.

Table S7 Predicted antimicrobial resistance phenotypes of the spinach metagenome assemblies

Sequencer	Assembler	Predicted antimicrobial resistance phenotype
HiSeq	ABySS	N.A.[a]
	IDBA-UD	Rifampicin, gentamicin, ciprofloxacin I/R, streptomycin, kanamycin, ampicillin, chloramphenicol, fosfomycin, amikacin, sulfisoxazole, tetracycline
	MaSuRCA	Streptomycin, kanamycin, fosfomycin, sulfisoxazole
	MEGAHIT	Rifampicin, gentamicin, ciprofloxacin I/R, streptomycin, kanamycin, ampicillin, ceftriaxone, amoxicillin/clavulanic acid, chloramphenicol, fosfomycin, amikacin, sulfisoxazole, tetracycline
	metaSPAdes	Rifampicin, gentamicin, ciprofloxacin I/R, streptomycin, hygromicin, kanamycin, ampicillin, ceftriaxone, amoxicillin/clavulanic acid, chloramphenicol, fosfomycin, amikacin, sulfisoxazole, tetracycline
	Ray Meta	Gentamicin, ciprofloxacin I/R, streptomycin, ampicillin, fosfomycin, sulfisoxazole
MiSeq	ABySS	Rifampicin, gentamicin, ciprofloxacin I/R, streptomycin, kanamycin, ampicillin, ceftriaxone, trimethoprim, chloramphenicol, fosfomycin, amikacin, sulfisoxazole, tetracycline, nalidixic acid
	IDBA-UD	Rifampicin, gentamicin, ciprofloxacin I/R, streptomycin, hygromicin, kanamycin, ampicillin, ceftriaxone, trimethoprim, fosfomycin, amikacin, sulfisoxazole, tetracycline, nalidixic acid
	MaSuRCA	Rifampicin, gentamicin, ciprofloxacin I/R, streptomycin, hygromicin, kanamycin, ampicillin, ceftriaxone, trimethoprim, chloramphenicol, fosfomycin, amikacin, sulfisoxazole, tetracycline, nalidixic acid
	MEGAHIT	Rifampicin, gentamicin, ciprofloxacin I/R, streptomycin, hygromicin, kanamycin, ampicillin, ceftriaxone, trimethoprim, chloramphenicol, fosfomycin, amikacin, sulfisoxazole, tetracycline, nalidixic acid
	metaSPAdes	Rifampicin, gentamicin, ciprofloxacin I/R, streptomycin, hygromicin, kanamycin, ampicillin, ceftriaxone, trimethoprim, chloramphenicol, fosfomycin, amikacin, sulfisoxazole, tetracycline, nalidixic acid
	Ray Meta	Rifampicin, streptomycin, hygromicin, kanamycin, ampicillin, ceftriaxone, ampicillin, fosfomycin, sulfisoxazole
NovaSeq	ABySS	Rifampicin, sulfisoxazole
	IDBA-UD	Rifampicin, gentamicin, ciprofloxacin I/R, streptomycin, hygromicin, kanamycin, ampicillin, ceftriaxone, trimethoprim, chloramphenicol, fosfomycin, amikacin, sulfisoxazole, tetracycline, nalidixic acid
	MaSuRCA	Gentamicin, streptomycin, kanamycin, trimethoprim, fosfomycin, amikacin, sulfisoxazole

	MEGAHIT	Rifampicin, gentamicin, ciprofloxacin I/R, streptomycin, hygromicin, kanamycin, ampicillin, ceftriaxone, trimethoprim, chloramphenicol, fosfomycin, amikacin, sulfisoxazole, tetracycline, nalidixic acid
	metaSPAdes	Rifampicin, gentamicin, ciprofloxacin I/R, streptomycin, hygromicin, kanamycin, ampicillin, ceftriaxone, trimethoprim, chloramphenicol, fosfomycin, amikacin, sulfisoxazole, tetracycline, nalidixic acid
	Ray Meta	Rifampicin, gentamicin, ciprofloxacin I/R, streptomycin, kanamycin, ampicillin, ceftriaxone, trimethoprim, fosfomycin, amikacin, sulfisoxazole
Reference		*S.* Indiana SI43: Rifampicin, gentamicin, ciprofloxacin I/R, streptomycin, hygromicin, kanamycin, ampicillin, ceftriaxone, trimethoprim, chloramphenicol, fosfomycin, amikacin, sulfisoxazole, tetracycline, nalidixic acid<br>*M. luteus* AS2: Chloramphenicol, sulfisoxazole<br>*Rhizobium* sp. S41: Streptomycin, kanamycin<br>*S. maltophilia* NCTC10258: Kanamycin

[a]N.A., not applicable.

Table S8 Antimicrobial resistance genotypes of the spinach metagenome assemblies with 1.5 million MiSeq reads[a]

Microorganism	Antimicrobial resistance gene identified in the reference	Assembler[b]					
		A	I	C	H	S	R
M. luteus AS2	*cmx*						
	sul1						
Rhizobium sp. S41	*aph(6)-Id*						
	strA						
S. maltophilia NCTC10258	*aph(3')-IIc*						
S. Indiana SI43	*ARR-3*						
	aac(3)-IId						
	aac(3)-IVa						
	aac(6')-Ib-cr						
	aac(6')-Ib-cr						
	aadA2						
	aph(3'')-Ib						
	aph(4)-Ia						
	aph(6)-Id						
	blaCTX-M-65						
	blaOXA-1						
	blaTEM-1B						
	catB4						
	dfrA12						
	floR						
	fosA3						
	rmtB						
	sul1						
	sul1						
	sul2						
	sul2						
	tet(A)						
	gyrA (D87N)						
	gyrA (S83F)						
	parC (S80R)						

[a]Green block indicates that the antimicrobial resistance gene was detected and confirmed to be originated from its corresponding reference genome, while yellow block indicates that it was detected but not confirmed to be originated from its corresponding reference genome and white block indicates that the antimicrobial resistance gene was not detected. Yellow block with a

cross indicates that *aph(6)-Id* or *sul1* was not confirmed to be originated from *S.* Indiana SI43 or other microorganisms.

[b]A, ABySS; I, IDBA-UD; C, MaSuRCA; H, MEGAHIT; S, metaSPAdes; R, Ray Meta.

Table S9 Additional antimicrobial resistance genes detected and those identified not to be in the reference genomes

Assembler	Antimicrobial resistance genes
ABySS	*sul1* (*S.* Indiana SI43), *sul2* (*S.* Indiana SI43)
MaSuRCA	*sul1* (*S.* Indiana SI43)

Table S10 Predicted antimicrobial resistance phenotypes of the spinach metagenome assemblies with 1.5 million MiSeq reads

Sequencer	Assembler	Predicted antimicrobial resistance phenotype
MiSeq	ABySS	Rifampicin, gentamicin, ciprofloxacin I/R, streptomycin, hygromicin, kanamycin, ampicillin, ceftriaxone, trimethoprim, chloramphenicol, fosfomycin, amikacin, sulfisoxazole, tetracycline, nalidixic acid
	IDBA-UD	Rifampicin, gentamicin, ciprofloxacin I/R, streptomycin, hygromicin, kanamycin, ampicillin, ceftriaxone, trimethoprim, chloramphenicol, fosfomycin, amikacin, sulfisoxazole, tetracycline, nalidixic acid
	MaSuRCA	Rifampicin, gentamicin, ciprofloxacin I/R, streptomycin, hygromicin, kanamycin, ampicillin, ceftriaxone, trimethoprim, chloramphenicol, fosfomycin, amikacin, sulfisoxazole, tetracycline, nalidixic acid
	MEGAHIT	Rifampicin, gentamicin, ciprofloxacin I/R, streptomycin, hygromicin, kanamycin, ampicillin, ceftriaxone, trimethoprim, chloramphenicol, fosfomycin, amikacin, sulfisoxazole, tetracycline, nalidixic acid
	metaSPAdes	Rifampicin, gentamicin, ciprofloxacin I/R, streptomycin, hygromicin, kanamycin, ampicillin, ceftriaxone, trimethoprim, chloramphenicol, fosfomycin, amikacin, sulfisoxazole, tetracycline, nalidixic acid
	Ray Meta	Rifampicin, gentamicin, ciprofloxacin I/R, streptomycin, hygromicin, kanamycin, ampicillin, ceftriaxone, trimethoprim, chloramphenicol, fosfomycin, amikacin, sulfisoxazole, tetracycline, nalidixic acid

Table S11 Antimicrobial resistance genotypes of the spinach metagenome assemblies with 2.4 million HiSeq reads and 2 million NovaSeq reads[a]

Microorganism	Antimicrobial resistance gene identified in the reference	Assembler[b]											
		HiSeq						NovaSeq					
		A	I	C	H	S	R	A	I	C	H	S	R
M. luteus AS2	*cmx*												
	sul1												
Rhizobium sp. S41	*aph(6)-Id*												
	strA												
S. maltophilia NCTC10258	*aph(3')-IIc*												
S. Indiana SI43	*ARR-3*												
	aac(3)-IId												
	aac(3)-IVa												
	aac(6')-Ib-cr												
	aac(6')-Ib-cr												
	aadA2												
	aph(3'')-Ib												
	aph(4)-Ia												
	aph(6)-Id												
	blaCTX-M-65												
	blaOXA-1												
	blaTEM-1B												
	catB4												
	dfrA12												
	floR												
	fosA3												
	rmtB												
	sul1												
	sul1												

	sul2												
	sul2												
	tet(A)												
	gyrA (D87N)												
	gyrA (S83F)												
	parC (S80R)												

[a]Green block indicates that the antimicrobial resistance gene was detected and confirmed to be originated from its corresponding reference genome, while yellow block indicates that it was detected but not confirmed to be originated from its corresponding reference genome and white block indicates that the antimicrobial resistance gene was not detected.

[b]A, ABySS; I, IDBA-UD; C, MaSuRCA; H, MEGAHIT; S, metaSPAdes; R, Ray Meta.

Table S12 Additional antimicrobial resistance genes detected and those identified not to be in the reference genomes

Sequencer	Assembler	Antimicrobial resistance genes
HiSeq	MaSuRCA	*sul2* (*S.* Indiana SI43)
NovaSeq	MaSuRCA	*bla*TEM-34, *sul1* (*S.* Indiana SI43)
	Ray Meta	*bla*TEM-30

[a]The antimicrobial resistance gene with an underline was not present in the reference genomes.

Table S13 Predicted antimicrobial resistance phenotypes of the spinach metagenome assemblies with 2.4 million HiSeq reads and 2 million NovaSeq reads

Sequencer	Assembler	Predicted antimicrobial resistance phenotype
HiSeq	ABySS	Rifampicin, gentamicin, ciprofloxacin I/R, streptomycin, hygromicin, kanamycin, ampicillin, ceftriaxone, trimethoprim, chloramphenicol, fosfomycin, amikacin, sulfisoxazole, tetracycline, nalidixic acid
	IDBA-UD	Rifampicin, gentamicin, ciprofloxacin I/R, streptomycin, hygromicin, kanamycin, ampicillin, ceftriaxone, trimethoprim, chloramphenicol, fosfomycin, amikacin, sulfisoxazole, tetracycline, nalidixic acid
	MaSuRCA	Rifampicin, gentamicin, ciprofloxacin I/R, streptomycin, hygromicin, kanamycin, ampicillin, ceftriaxone, chloramphenicol, amikacin, sulfisoxazole, tetracycline, nalidixic acid
	MEGAHIT	Rifampicin, gentamicin, ciprofloxacin I/R, streptomycin, hygromicin, kanamycin, ampicillin, ceftriaxone, trimethoprim, chloramphenicol, fosfomycin, amikacin, sulfisoxazole, tetracycline, nalidixic acid
	metaSPAdes	Rifampicin, gentamicin, ciprofloxacin I/R, streptomycin, hygromicin, kanamycin, ampicillin, ceftriaxone, trimethoprim, chloramphenicol, fosfomycin, amikacin, sulfisoxazole, tetracycline, nalidixic acid
	Ray Meta	Rifampicin, gentamicin, ciprofloxacin I/R, streptomycin, hygromicin, kanamycin, ampicillin, ceftriaxone, trimethoprim, chloramphenicol, fosfomycin, amikacin, sulfisoxazole, tetracycline
NovaSeq	ABySS	Rifampicin, gentamicin, ciprofloxacin I/R, streptomycin, hygromicin, kanamycin, ampicillin, ceftriaxone, trimethoprim, chloramphenicol, fosfomycin, amikacin, sulfisoxazole, tetracycline, nalidixic acid
	IDBA-UD	Rifampicin, gentamicin, ciprofloxacin I/R, streptomycin, hygromicin, kanamycin, ampicillin, ceftriaxone, trimethoprim, chloramphenicol, fosfomycin, amikacin, sulfisoxazole, tetracycline, nalidixic acid
	MaSuRCA	Rifampicin, gentamicin, ciprofloxacin I/R, streptomycin, hygromicin, kanamycin, ampicillin, ceftriaxone, trimethoprim, amoxicillin/clavulanic acid, chloramphenicol, fosfomycin, amikacin, sulfisoxazole, tetracycline, nalidixic acid
	MEGAHIT	Rifampicin, gentamicin, ciprofloxacin I/R, streptomycin, hygromicin, kanamycin, ampicillin, ceftriaxone, trimethoprim, chloramphenicol, fosfomycin, amikacin, sulfisoxazole, tetracycline, nalidixic acid
	metaSPAdes	Rifampicin, gentamicin, ciprofloxacin I/R, streptomycin, hygromicin, kanamycin, ampicillin, ceftriaxone, trimethoprim,

	chloramphenicol, fosfomycin, amikacin, sulfisoxazole, tetracycline, nalidixic acid
Ray Meta	Rifampicin, gentamicin, ciprofloxacin I/R, streptomycin, hygromicin, kanamycin, ampicillin, ceftriaxone, amoxicillin/clavulanic acid, trimethoprim, chloramphenicol, fosfomycin, amikacin, sulfisoxazole, tetracycline

Table S14 Antimicrobial resistance genotypes of the surface water metagenome assemblies with 1.5 million MiSeq reads[a]

Microorganism	Antimicrobial resistance gene identified in the reference	Assembler[b]					
		A	I	C	H	S	R
P. norimbergensis DSM 11628	*blaOXA-157*		■		■	■	
S. erythraea NRRL 2338	*erm(E)*		■	■		■	
P. aeruginosa PAO1	*aph(3')-IIb*						
	blaOXA-50						
	blaPAO			■		■	
	catB7		■	■		■	
	fosA		■	■		■	

[a]Green block indicates that the antimicrobial resistance gene was detected and confirmed to be originated from its corresponding reference genome, while white block indicates that the antimicrobial resistance gene was not detected.

[b]A, ABySS; I, IDBA-UD; C, MaSuRCA; H, MEGAHIT; S, metaSPAdes; R, Ray Meta.

Table S15 Predicted antimicrobial resistance phenotypes of the spinach metagenome assemblies with 1.5 million MiSeq reads

Sequencer	Assembler	Predicted antimicrobial resistance phenotype
MiSeq	ABySS	N.A.[a]
	IDBA-UD	Ampicillin, amoxicillin/clavulanic acid, cefoxitin, ceftriaxone, meropenem, chloramphenicol, erythromycin, azithromycin, fosfomycin
	MaSuRCA	N.A.
	MEGAHIT	Ampicillin, amoxicillin/clavulanic acid, cefoxitin, ceftriaxone, meropenem, chloramphenicol, erythromycin, azithromycin, fosfomycin
	metaSPAdes	Ampicillin, amoxicillin/clavulanic acid, cefoxitin, ceftriaxone, meropenem, chloramphenicol, erythromycin, azithromycin, fosfomycin
	Ray Meta	N.A.
Reference	*P. aeruginosa* PAO1: Kanamycin, ampicillin, amoxicillin/clavulanic acid, cefoxitin, ceftriaxone, chloramphenicol, fosfomycin	
	P. norimbergensis DSM 11628: Ampicillin, amoxicillin/clavulanic acid, cefoxitin, ceftriaxone, meropenem	
	S. erythraea NRRL 2338: Erythromycin, azithromycin	

[a]N.A., not applicable.

Table S16 Antimicrobial resistance genotypes of the surface water metagenome assemblies with 2.4 million HiSeq reads and two million NovaSeq reads[a]

Microorganism	Antimicrobial resistance gene identified in the reference	Assembler[b]											
		HiSeq						NovaSeq					
		A	I	C	H	S	R	A	I	C	H	S	R
P. norimbergensis DSM 11628	*blaOXA-157*												
S. erythraea NRRL 2338	*erm(E)*												
P. aeruginosa PAO1	*aph(3')-IIb*												
	blaOXA-50												
	blaPAO												
	catB7												
	fosA												

[a]Green block indicates that the antimicrobial resistance gene was detected and confirmed to be originated from its corresponding reference genome, while yellow block indicates that it was detected but not confirmed to be originated from its corresponding reference genome and white block indicates that the antimicrobial resistance gene was not detected.

[b]A, ABySS; I, IDBA-UD; C, MaSuRCA; H, MEGAHIT; S, metaSPAdes; R, Ray Meta.

Table S17 Predicted antimicrobial resistance phenotypes of the surface water metagenome assemblies with 2.4 million HiSeq reads and two million NovaSeq reads

Sequencer	Assembler	Predicted antimicrobial resistance phenotype
HiSeq	ABySS	N.A.[a]
	IDBA-UD	N.A.
	MaSuRCA	N.A.
	MEGAHIT	N.A.
	metaSPAdes	Kanamycin, fosfomycin
	Ray Meta	N.A.
NovaSeq	ABySS	N.A.
	IDBA-UD	Ampicillin, amoxicillin/clavulanic acid, cefoxitin, ceftriaxone, meropenem
	MaSuRCA	Ampicillin, amoxicillin/clavulanic acid, cefoxitin, ceftriaxone, meropenem
	MEGAHIT	Ampicillin, amoxicillin/clavulanic acid, cefoxitin, ceftriaxone, meropenem, erythromycin, azithromycin
	metaSPAdes	Ampicillin, amoxicillin/clavulanic acid, cefoxitin, ceftriaxone, meropenem
	Ray Meta	N.A.

[a]N.A., not applicable.

Table S18 Antimicrobial resistance genotypes of the surface water metagenome assemblies with 4.8 million HiSeq reads, two million MiSeq reads, and four million NovaSeq reads[a]

Microorganism	Antimicrobial resistance gene identified in the reference	Assembler[b]																	
		HiSeq						MiSeq						NovaSeq					
		A	I	C	H	S	R	A	I	C	H	S	R	A	I	C	H	S	R
P. norimbergensis DSM 11628	*blaOXA-157*		[green]		[green]	[green]			[green]		[green]	[green]			[green]		[green]	[green]	
S. erythraea NRRL 2338	*erm(E)*		[green]		[green]	[green]			[green]		[green]	[green]		[green]	[green]	[green]	[green]	[green]	[yellow]
P. aeruginosa PAO1	*aph(3')-IIb*											[green]		[green]	[green]	[green]	[green]	[green]	
	blaOXA-50								[green]		[green]			[green]	[green]	[green]	[green]	[green]	
	blaPAO								[green]										
	catB7											[green]		[green]	[green]	[green]	[green]	[green]	
	fosA	[green]	[green]		[green]				[green]		[green]	[green]						[green]	

[a]Green block indicates that the antimicrobial resistance gene was detected and confirmed to be originated from its corresponding reference genome, while yellow block indicates that it was detected but not confirmed to be originated from its corresponding reference genome and white block indicates that the antimicrobial resistance gene was not detected.

[b]A, ABySS; I, IDBA-UD; C, MaSuRCA; H, MEGAHIT; S, metaSPAdes; R, Ray Meta.

Table S19 Predicted antimicrobial resistance phenotypes of the surface water metagenome assemblies with 4.8 million HiSeq reads, two million MiSeq reads, and four million NovaSeq reads

Sequencer	Assembler	Predicted antimicrobial resistance phenotype
HiSeq	ABySS	Fosfomycin
	IDBA-UD	Ampicillin, amoxicillin/clavulanic acid, cefoxitin, ceftriaxone, meropenem, erythromycin, azithromycin, fosfomycin
	MaSuRCA	N.A.[a]
	MEGAHIT	Ampicillin, amoxicillin/clavulanic acid, cefoxitin, ceftriaxone, meropenem, erythromycin, azithromycin, fosfomycin
	metaSPAdes	Ampicillin, amoxicillin/clavulanic acid, cefoxitin, ceftriaxone, meropenem, erythromycin, azithromycin
	Ray Meta	N.A.
MiSeq	ABySS	N.A.
	IDBA-UD	Ampicillin, amoxicillin/clavulanic acid, cefoxitin, ceftriaxone, meropenem, erythromycin, azithromycin, fosfomycin
	MaSuRCA	N.A.
	MEGAHIT	Ampicillin, amoxicillin/clavulanic acid, cefoxitin, ceftriaxone, meropenem, erythromycin, azithromycin, fosfomycin
	metaSPAdes	Kanamycin, ampicillin, amoxicillin/clavulanic acid, cefoxitin, ceftriaxone, meropenem, chloramphenicol, erythromycin, azithromycin, fosfomycin
	Ray Meta	N.A.
NovaSeq	ABySS	Kanamycin, ampicillin, chloramphenicol, erythromycin, azithromycin
	IDBA-UD	Kanamycin, ampicillin, amoxicillin/clavulanic acid, cefoxitin, ceftriaxone, meropenem, chloramphenicol, erythromycin, azithromycin
	MaSuRCA	Kanamycin, ampicillin, chloramphenicol, erythromycin, azithromycin
	MEGAHIT	Kanamycin, ampicillin, amoxicillin/clavulanic acid, cefoxitin, ceftriaxone, meropenem, chloramphenicol, erythromycin, azithromycin
	metaSPAdes	Kanamycin, ampicillin, amoxicillin/clavulanic acid, cefoxitin, ceftriaxone, meropenem, chloramphenicol, erythromycin, azithromycin, fosfomycin
	Ray Meta	Erythromycin, azithromycin

[a]N.A., not applicable.

Table S20 Antimicrobial resistance genotypes of the reads classified as *Salmonella* in the spinach metagenome assemblies with one million reads[a]

Antimicrobial resistance gene identified in the reference	Assembler[b]																	
	HiSeq						MiSeq						NovaSeq					
	A	I	C	H	S	R	A	I	C	H	S	R	A	I	C	H	S	R
ARR-3																		
aac(3)-IId																		
aac(3)-IVa																		
aac(6')-Ib-cr																		
aac(6')-Ib-cr																		
aadA2																		
aph(3'')-Ib																		
aph(4)-Ia																		
aph(6)-Id																		
blaCTX-M-65																		
blaOXA-1																		
blaTEM-1B																		
catB4																		
dfrA12																		
floR																		
fosA3																		
rmtB																		
sul1																		
sul1																		
sul2																		
sul2																		
tet(A)																		
gyrA (D87N)																		
gyrA (S83F)																		

parC (S80R)																		

[a]Green block indicates that the antimicrobial resistance gene was detected and confirmed to be originated from its corresponding reference genome, while the lightest green block indicates that the antimicrobial resistance gene was not detected.

[b]A, ABySS; I, IDBA-UD; C, MaSuRCA; H, MEGAHIT; S, metaSPAdes; R, Ray Meta.

Table S21 Predicted antimicrobial resistance phenotypes of the reads classified as *Salmonella* in the spinach metagenome assemblies with one million reads

Sequencer	Assembler	Predicted antimicrobial resistance phenotype
HiSeq	ABySS	N.A.[a]
	IDBA-UD	Streptomycin, kanamycin
	MaSuRCA	Streptomycin, kanamycin, sulfisoxazole
	MEGAHIT	N.A.
	metaSPAdes	N.A.
	Ray Meta	N.A.
MiSeq	ABySS	N.A.
	IDBA-UD	N.A.
	MaSuRCA	N.A.
	MEGAHIT	Sulfisoxazole
	metaSPAdes	N.A.
	Ray Meta	N.A.
NovaSeq	ABySS	N.A.
	IDBA-UD	N.A.
	MaSuRCA	Sulfisoxazole
	MEGAHIT	N.A.
	metaSPAdes	N.A.
	Ray Meta	Sulfisoxazole

[a]N.A., not applicable.

Tables S22 Antimicrobial resistance genotypes of the reads classified as *Salmonella* in the spinach metagenome assemblies with 1.5 million MiSeq reads

Microorganism	Antimicrobial resistance gene identified in the reference	Assembler[b]					
		MiSeq					
		A	I	C	H	S	R
M. luteus AS2	*cmx*						
	sul1						
Rhizobium sp. S41	*aph(6)-Id*						
	strA						
S. maltophilia NCTC10258	*aph(3')-IIc*						
S. Indiana SI43	*ARR-3*						
	aac(3)-IId						
	aac(3)-IVa						
	aac(6')-Ib-cr						
	aac(6')-Ib-cr						
	aadA2						
	aph(3'')-Ib						
	aph(4)-Ia						
	aph(6)-Id						
	blaCTX-M-65						
	blaOXA-1						
	blaTEM-1B						
	catB4						
	dfrA12						
	floR						
	fosA3						
	rmtB						
	sul1						
	sul1						
	sul2						
	sul2						
	tet(A)						
	gyrA (D87N)						
	gyrA (S83F)						
	parC (S80R)						

[a]Green block indicates that the antimicrobial resistance gene was detected and confirmed to be originated from its corresponding reference genome, while the lightest green block indicates that the antimicrobial resistance gene was not detected.

[b]A, ABySS; I, IDBA-UD; C, MaSuRCA; H, MEGAHIT; S, metaSPAdes; R, Ray Meta.

Tables S23 Predicted antimicrobial resistance phenotypes of the reads classified as *Salmonella* in the spinach metagenome assemblies with 1.5 million MiSeq reads

Sequencer	Assembler	Predicted antimicrobial resistance phenotype
MiSeq	ABySS	N.A.[a]
	IDBA-UD	Streptomycin, kanamycin
	MaSuRCA	Gentamicin, hygromicin, ampicillin, ceftriaxone, sulfisoxazole
	MEGAHIT	Streptomycin, kanamycin
	metaSPAdes	N.A.
	Ray Meta	Streptomycin, kanamycin, sulfisoxazole

[a]N.A., not applicable.

Tables S24 Antimicrobial resistance genotypes of the reads classified as *Salmonella* in the spinach metagenome assemblies with 2.4 million HiSeq reads and 2 million NovaSeq reads[a]

Antimicrobial resistance gene identified in the reference	Assembler[b]											
	HiSeq						NovaSeq					
	A	I	C	H	S	R	A	I	C	H	S	R
ARR-3												
aac(3)-IId												
aac(3)-IVa												
aac(6')-Ib-cr												
aac(6')-Ib-cr												
aadA2												
aph(3'')-Ib												
aph(4)-Ia												
aph(6)-Id												
blaCTX-M-65												
blaOXA-1												
blaTEM-1B												
catB4												
dfrA12												
floR												
fosA3												
rmtB												
sul1												
sul1												
sul2												
sul2												
tet(A)												
gyrA (D87N)												
gyrA (S83F)												

parC (S80R)												

[a]Green block indicates that the antimicrobial resistance gene was detected and confirmed to be originated from its corresponding reference genome, while lighter green block indicates that it was detected but not confirmed to be originated from its corresponding reference genome and the lightest green block indicates that the antimicrobial resistance gene was not detected. Lighter green block with a cross indicates that *aph(6)-Id* or *sul1* was not confirmed to be originated from *S.* Indiana SI43 or other microorganisms.

[b]A, ABySS; I, IDBA-UD; C, MaSuRCA; H, MEGAHIT; S, metaSPAdes; R, Ray Meta.

Tables S25 Predicted antimicrobial resistance phenotypes of the reads classified as *Salmonella* in the spinach metagenome assemblies with 2.4 million HiSeq reads and 2 million NovaSeq reads

Sequencer	Assembler	Predicted antimicrobial resistance phenotype
HiSeq	ABySS	Sulfisoxazole
	IDBA-UD	N.A.[a]
	MaSuRCA	Gentamicin, hygromicin, sulfisoxazole
	MEGAHIT	Streptomycin, kanamycin
	metaSPAdes	N.A.
	Ray Meta	N.A.
NovaSeq	ABySS	Streptomycin, kanamycin, sulfisoxazole
	IDBA-UD	N.A.
	MaSuRCA	N.A.
	MEGAHIT	Streptomycin, kanamycin
	metaSPAdes	N.A.
	Ray Meta	Sulfisoxazole

[a]N.A., not applicable.

Table S26 Antimicrobial resistance genotypes of the reads classified as *Pseudomonas* in the surface water metagenome assemblies with 1.5 million MiSeq reads[a]

Microorganism	Antimicrobial resistance gene identified in the reference	Assembler[b]					
		A	I	C	H	S	R
P. norimbergensis DSM 11628	*blaOXA-157*						
S. erythraea NRRL 2338	*erm(E)*						
P. aeruginosa PAO1	*aph(3')-IIb*						
	blaOXA-50						
	blaPAO						
	catB7						
	fosA						

[a]Green block indicates that the antimicrobial resistance gene was detected and confirmed to be originated from its corresponding reference genome, while the lightest green block indicates that the antimicrobial resistance gene was not detected.

[b]A, ABySS; I, IDBA-UD; C, MaSuRCA; H, MEGAHIT; S, metaSPAdes; R, Ray Meta.

Table S27 Antimicrobial resistance genotypes of the reads classified as *Pseudomonas* in the surface water metagenome assemblies with 2.4 million HiSeq reads and 2 million NovaSeq reads[a]

Microorganism	Antimicrobial resistance gene identified in the reference	Assembler[b]											
		HiSeq						NovaSeq					
		A	I	C	H	S	R	A	I	C	H	S	R
P. norimbergensis DSM 11628	*blaOXA-157*												
S. erythraea NRRL 2338	*erm(E)*												
P. aeruginosa PAO1	*aph(3')-IIb*												
	blaOXA-50												
	blaPAO												
	catB7												
	fosA												

[a]Green block indicates that the antimicrobial resistance gene was detected and confirmed to be originated from its corresponding reference genome, while the lightest green block indicates that the antimicrobial resistance gene was not detected.

[b]A, ABySS; I, IDBA-UD; C, MaSuRCA; H, MEGAHIT; S, metaSPAdes; R, Ray Meta.

Table S28 Predicted antimicrobial resistance phenotypes of the reads classified as *Pseudomonas* in the surface water metagenome assemblies with 2.4 million HiSeq reads and 2 million NovaSeq reads

Sequencer	Assembler	Predicted antimicrobial resistance phenotype
NovaSeq	metaSPAdes	Ampicillin

Table S29 Antimicrobial resistance genotypes of the reads classified as *Pseudomonas* in the surface water metagenome assemblies with 4.8 million HiSeq reads, two million MiSeq reads, and four million NovaSeq reads[a]

Microorganism	Antimicrobial resistance gene identified in the reference	Assembler[b]																	
		HiSeq						MiSeq						NovaSeq					
		A	I	C	H	S	R	A	I	C	H	S	R	A	I	C	H	S	R
P. norimbergensis DSM 11628	*blaOXA-157*																		
S. erythraea NRRL 2338	*erm(E)*																		
P. aeruginosa PAO1	*aph(3')-IIb*																		
	blaOXA-50																		
	blaPAO																		
	catB7																		
	fosA																		

[a]Green block indicates that the antimicrobial resistance gene was detected and confirmed to be originated from its corresponding reference genome, while the lightest green block indicates that the antimicrobial resistance gene was not detected.

[b]A, ABySS; I, IDBA-UD; C, MaSuRCA; H, MEGAHIT; S, metaSPAdes; R, Ray Meta.

Table S30 Predicted antimicrobial resistance phenotypes of the reads classified as *Pseudomonas* in the surface water metagenome assemblies with 4.8 million HiSeq reads, two million MiSeq reads, and four million NovaSeq reads

Sequencer	Assembler	Predicted antimicrobial resistance phenotype
NovaSeq	ABySS	Ampicillin
	IDBA-UD	Ampicillin
	MEGAHIT	Ampicillin

Table S31 Numbers of virulence genes in the reads classified as *Salmonella* in the spinach metagenome assemblies

Sequencing depth (million)	Sequencer	Assembler					
		ABySS	IDBA-UD	MaSuRCA	MEGAHIT	metaSPAdes	Ray Meta
1	HiSeq	0	9	5	26	6	17
	MiSeq	1	2	0	0	0	32
	NovaSeq	7	2	9	12	0	30
2.4	HiSeq	112	122	105	123	126	100
2	NovaSeq	111	124	105	124	126	97
1.5	MiSeq	0	0	0	0	0	0

Table S32 Numbers of virulence genes in the reads classified as *Pseudomonas* in the surface water metagenome assemblies

Sequencing depth (million)	Sequencer	Assembler					
		ABySS	IDBA-UD	MaSuRCA	MEGAHIT	metaSPAdes	Ray Meta
2.4	HiSeq	1	3	1	6	19	0
1.5	MiSeq	0	17	0	24	17	5
2	NovaSeq	0	6	1	11	25	1
4.8	HiSeq	2	17	N.A.[a]	29	16	6
2	MiSeq	3	15	2	30	14	6
4	NovaSeq	9	25	2	32	23	6

[a]N.A., not applicable.

Table S33 *Salmonella* pathogenicity island (SPI) typing of the reads classified as *Salmonella* in the spinach metagenome assemblies

Sequencing depth (million)	Sequencer	Assembler						Reference
		ABySS	IDBA-UD	MaSuRCA	MEGAHIT	metaSPAdes	Ray Meta	
1	HiSeq	N.D.[a]	SPI-1 (2)	SPI-2 (2)	SPI-1 (5)	SPI-1 (3)	SPI-2 (2)	SPI-1 (8)<br>SPI-2 (6)<br>SPI-3 (3)<br>SPI-4 (1)<br>SPI-5 (1)<br>SPI-9 (1)
	MiSeq	N.D.	N.D.	N.D.	N.D.	N.D.	SPI-1 (2)<br>SPI-2 (2)	
	NovaSeq	N.D.	N.D.	SPI-2 (1)	SPI-1 (1)	N.D.	SPI-1 (3)<br>SPI-2 (1)	
2.4	HiSeq	N.D.[a]	N.D.	N.D.	N.D.	N.D.	SPI-1 (2)	
2	NovaSeq	N.D.	N.D.	N.D.	N.D.	N.D.	SPI-1 (2)<br>SPI-2 (1)	
1.5	MiSeq	SPI-1 (8)<br>SPI-2 (6)<br>SPI-3 (3)<br>SPI-4 (1)<br>SPI-5 (1)<br>SPI-9 (1)	SPI-1 (8)<br>SPI-2 (6)<br>SPI-3 (3)<br>SPI-5 (1)<br>SPI-9 (1)	SPI-1 (8)<br>SPI-2 (6)<br>SPI-3 (3)<br>SPI-4 (1)<br>SPI-5 (1)<br>SPI-9 (1)	SPI-1 (8)<br>SPI-2 (6)<br>SPI-3 (2)<br>SPI-5 (1)<br>SPI-9 (1)	SPI-1 (8)<br>SPI-2 (6)<br>SPI-3 (3)<br>SPI-4 (1)<br>SPI-5 (1)<br>SPI-9 (1)	SPI-1 (8)<br>SPI-2 (5)<br>SPI-3 (3)<br>SPI-5 (1)<br>SPI-9 (1)	

[a]N.D.not detectable.

Table S34 *Salmonella* serotyping of the reads classified as *Salmonella* in the spinach metagenome assemblies

Sequencing depth (million)	Sequencer	Assembler					
		ABySS	IDBA-UD	MaSuRCA	MEGAHIT	metaSPAdes	Ray Meta
1	HiSeq	Indiana	Indiana	Indiana	Indiana	Indiana	Indiana
	MiSeq	Indiana	Senftenberg	N.T.[a]	Indiana	Senftenberg	Indiana
	NovaSeq	Indiana	Indiana	Indiana	Indiana	Indiana	Indiana
2.4	HiSeq	Senftenberg	Senftenberg	N.T.[a]	Indiana	Senftenberg	Indiana
2	NovaSeq	Senftenberg	Senftenberg	Senftenberg	Senftenberg	Senftenberg	Indiana
1.5	MiSeq	Senftenberg	Senftenberg	Senftenberg	Cerro	Senftenberg	Indiana

[a]N.T., not typable.

Table S35 Multilocus sequence typing (MLST) of the reads classified as *Salmonella* in the spinach metagenome assemblies

Sequencing depth (million)	Sequencer	Assembler					
		ABySS	IDBA-UD	MaSuRCA	MEGAHIT	metaSPAdes	Ray Meta
1	HiSeq	N.T.[a]	N.T.	N.T.	*E. coli*	N.T.	*S. enterica*
	MiSeq	N.T.	N.T.	N.T.	N.T.	N.T.	*S. enterica*
	NovaSeq	N.T.	N.T.	N.T.	N.T.	N.T.	N.T.
2.4	HiSeq	N.T.[a]	N.T.	N.T.	*E. coli*	N.T.	*S. enterica*
2	NovaSeq	N.T.	N.T.	N.T.	N.T.	N.T.	N.T.
1.5	MiSeq	N.T.[a]	N.T.	N.T.	N.T.	N.T.	N.T.

[a]N.T., not typable.
